# Robust kinetics estimation from kinematics via direct collocation

**DOI:** 10.3389/fbioe.2024.1483225

**Published:** 2024-12-18

**Authors:** Kuan Wang, Linlin Zhang, Leichao Liang, Jiang Shao, Xinpeng Chen, Huihao Wang

**Affiliations:** ^1^ College of Rehabilitation Sciences, Shanghai University of Medicine and Health Sciences, Shanghai, China; ^2^ YangZhi Rehabilitation Hospital (Shanghai Sunshine Rehabilitation Center), School of Medicine, Tongji University, Shanghai, China; ^3^ Shi’s Center of Orthopedics and Traumatology (Institute of Traumatology, Shuguang Hospital), Shuguang Hospital Affiliated to Shanghai University of Traditional Chinese Medicine, Shanghai, China

**Keywords:** kinetics, kinematics, ground reaction force, direct collocation, simulation

## Abstract

**Introduction:**

Accurate joint moment analysis is essential in biomechanics, and the integration of direct collocation with markerless motion capture offers a promising approach for its estimation. However, markerless motion capture can introduce varying degrees of error in tracking trajectories. This study aims to evaluate the effectiveness of the direct collocation method in estimating kinetics when joint trajectory data are impacted by noise.

**Methods:**

We focused on walking and squatting movements as our target activities. To assess the method's robustness, we created five groups with differing noise levels—noise-free, mild noise, noisy group1, noisy group2, and a Gaussian noise group—in the joint center trajectories. Our approach involved combining joint center tracking with biological terms within the direct collocation scheme to address noise-related challenges. We calculated kinematics, joint moments, and ground reaction forces for comparison across the different noise groups.

**Results:**

For the walking task, the mean absolute errors (MAEs) for the knee flexion moments were 0.103, 0.113, 0.127, 0.129, and 0.116 Nm/kg across the respective noise levels. The corresponding MAEs of the ankle flexion moment were 0.130, 0.133, 0.145, 0.131, and 0.138 Nm/kg. The hip flexion moment had MAEs of 0.182, 0.204, 0.242, 0.246, and 0.249 Nm/kg in the respective groups. In squatting, the MAEs of ankle flexion moments were 0.207, 0.219, 0.217, 0.253, and 0.227 Nm/kg in the noise-free, mild noise, noisy group1, noisy group2, and the Gaussian noise group, respectively. The MAEs of the knee flexion moments were 0.177, 0.196, 0.198, 0.197, and 0.221 Nm/kg, whereas the mean MAEs of the hip flexion moments were 0.125, 0.135, 0.141, 0.161, and 0.178 Nm/kg in the respective groups.

**Conclusion:**

The results highlight that the direct collocation method incorporating both tracking and biological terms in the cost function could robustly estimate joint moments during walking and squatting across various noise levels. Currently, this method is better suited to reflect general activity dynamics than subject-specific dynamics in clinical practice. Future research should focus on refining cost functions to achieve an optimal balance between robustness and accuracy.

## Introduction

Joint moment analysis is crucial in biomechanics because it provides insights into the forces and torques acting on joints during movement. Understanding these moments is essential for exploring movement mechanics, which is helpful for performance optimization and ergonomics (Dos’Santos et al., 2021; Kim et al., 2024). Joint moment analysis is also a powerful tool in biomechanics that aids in injury prevention and rehabilitation and contributes to the design of better prosthetic and orthotic devices (Rogers-Bradley et al., 2024; Ye et al., 2024).

To acquire the joint moment during various movements, inverse dynamics is often used as a computational technique to calculate the forces and moments at the joints of a biomechanical system based on observed motion. By leveraging kinematic data (positions, velocities, and accelerations) from body segments and applying Newton’s laws of motion, inverse dynamics can be used to determine the net forces and moments necessary to produce observed movements (Ojeda et al., 2016). However, this method requires external force measurements (e.g., ground reaction forces) and relies on accurate kinematics obtained from motion capture systems and inertial parameters in musculoskeletal modeling. Unfortunately, the high cost and in-laboratory setup of motion capture systems and force plates limit the application of inverse dynamics in real-world scenarios.

With advances in deep learning, pose estimation technology has become an alternative to optical motion capture systems and has achieved acceptable accuracy (Wren et al., 2023; Outerleys et al., 2024). Markerless motion capture, also known as pose estimation, is a computer vision task that involves detecting and tracking the position and orientation of human body parts in images or videos. By predicting specific keypoints (such as joints, hands, and heads), pose estimation creates a skeletal representation of the subject. Notably, this approach is cost-effective and easily adaptable to various environments. Recently, pose estimation algorithms have become increasingly popular for analyzing human movement and understanding the mechanics of the body (Berhouet and Samargandi, 2024; Simonet et al., 2024). For example, pose estimation aids in the early diagnosis of movement disorders in patients with Parkinson’s disease (Hong et al., 2022). Athletes’ movements can be analyzed to improve techniques and prevent injuries (Monteiro et al., 2024). Pose estimation can also be used as a tool for physiotherapists to monitor patients’ ability to perform functional movements of the lower limbs (Hu et al., 2021). However, it is essential to consider potential errors arising from the number of cameras and the design of pose estimation algorithms, particularly when estimating joint centers (Wade et al., 2022).

Similar to motion capture systems, force plates are also essential for traditional biomechanics analysis in measuring the ground reaction force (GRF) for inverse dynamics calculations. Recently, optimal control has offered an alternative to sensor-based GRF measurements for tracking and simulation (Moissenet et al., 2019). By introducing contact elements in the musculoskeletal model, the contact dynamics are transformed into a differentiable and optimizable problem. In recent years, direct collocation has gained popularity as a method of optimal control in biomechanics (Lin and Pandy, 2017; Uhlrich et al., 2023; D’Hondt et al., 2024). This technique discretizes control and state variables at specific collocation points, effectively transforming the continuous-time optimal control problem into a finite-dimensional nonlinear programming problem. The key advantage lies in its simultaneous optimization of state and control trajectories, resulting in faster convergence than other approaches, such as direct single shooting (Betts, 2010; Porsa et al., 2016).

Widely employed to estimate muscle forces and joint moments, the direct collocation method has been successfully used to simulate various activities (Uhlrich et al., 2023). For example, given kinematics and GRFs, Lin and Pandy (2017) applied direct collocation to track joint angles and GRFs in musculoskeletal simulations. Falisse et al. (2019b) developed a unique cost function to ensure biological plausibility in predictive walking simulations without direct tracking of ground truth kinematics. Despite its potential, the ability of direct collocation to track joint centers has not been extensively examined, which is crucial given the variable errors introduced by different markerless motion capture setups.

A key distinction between tracking and predictive simulations in direct collocation lies in cost function design. To address the impact of noisy data, our study explored the combination of joint center tracking terms with biological terms used in predictive simulations. Our aim is to evaluate the effectiveness of this combination in estimating kinetics when joint trajectory inputs are noisy. We hypothesize that incorporating these terms into the cost function will increase the robustness of the kinetics estimation while tracking noisy joint center trajectories.

## Methods

**FIGURE 1:**
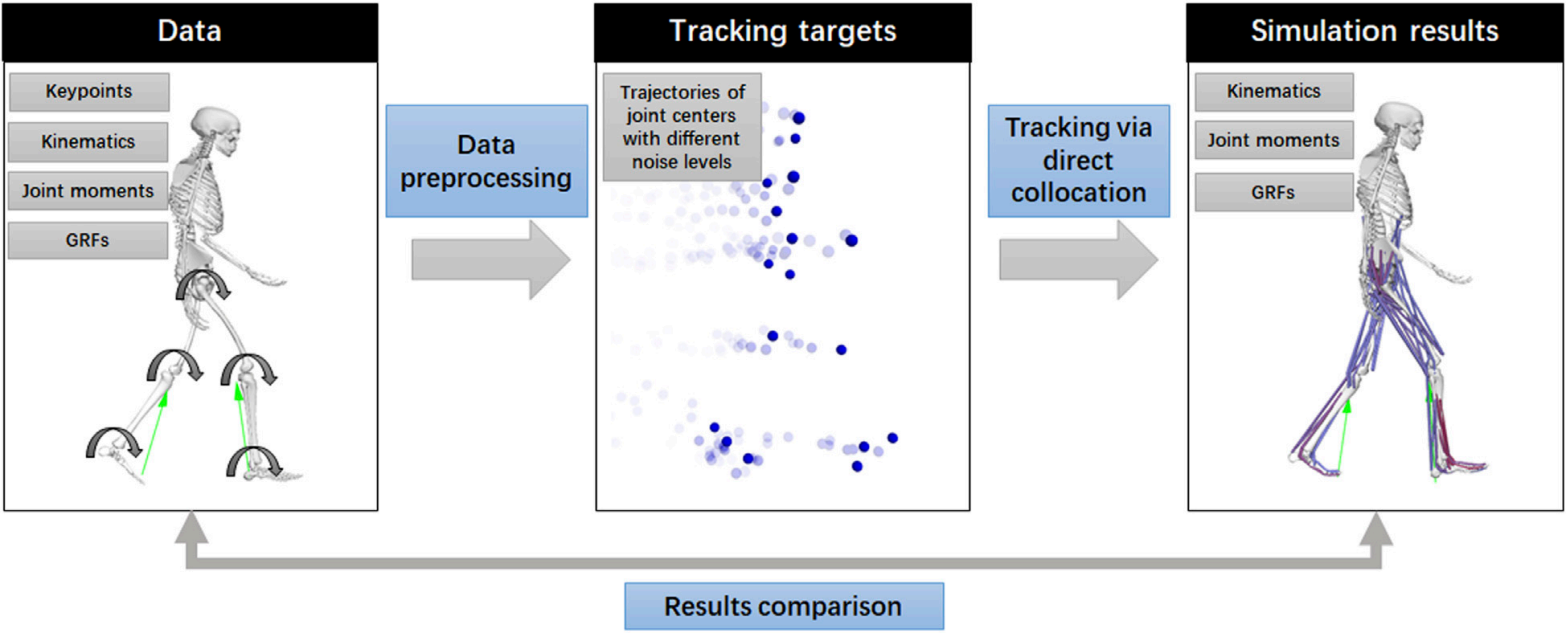
Data processing steps in this study.

The analysis comprises three essential processes: raw data preprocessing, joint center tracking via direct collocation, and result comparison (Figure 1).

To evaluate the capability of kinetics estimation, ground truth kinematics, GRFs, and joint moments are needed. Since walking represents basic human movement, the OpenCap biomechanics data on walking were used for analysis (Uhlrich et al., 2023). The extracted walking data included 10 able-bodied adults walking at their preferred speed. This dataset included full-body kinematics and GRFs captured via optical motion capture systems with ground-embedded force plates. Joint moments were also included in the dataset and were calculated based on the kinematics and GRFs using the OpenSim’s inverse dynamics function (Seth et al., 2018). The marker-based data, GRFs, and joint moments were used as the ground truth for comparison. Additionally, we analyzed squatting activity in the OpenCap dataset because of its unique role in fitness and daily activities. Similar to the walking dataset, this dataset provides full-body kinematics, GRFs, and joint moments. The study participants consisted of 10 healthy adults, including six female and four male candidates, with an average age of 27.7 years (±3.8), an average mass of 69.2 kg (±11.6), and an average height of 1.74 m (±0.12).

**FIGURE 2:**
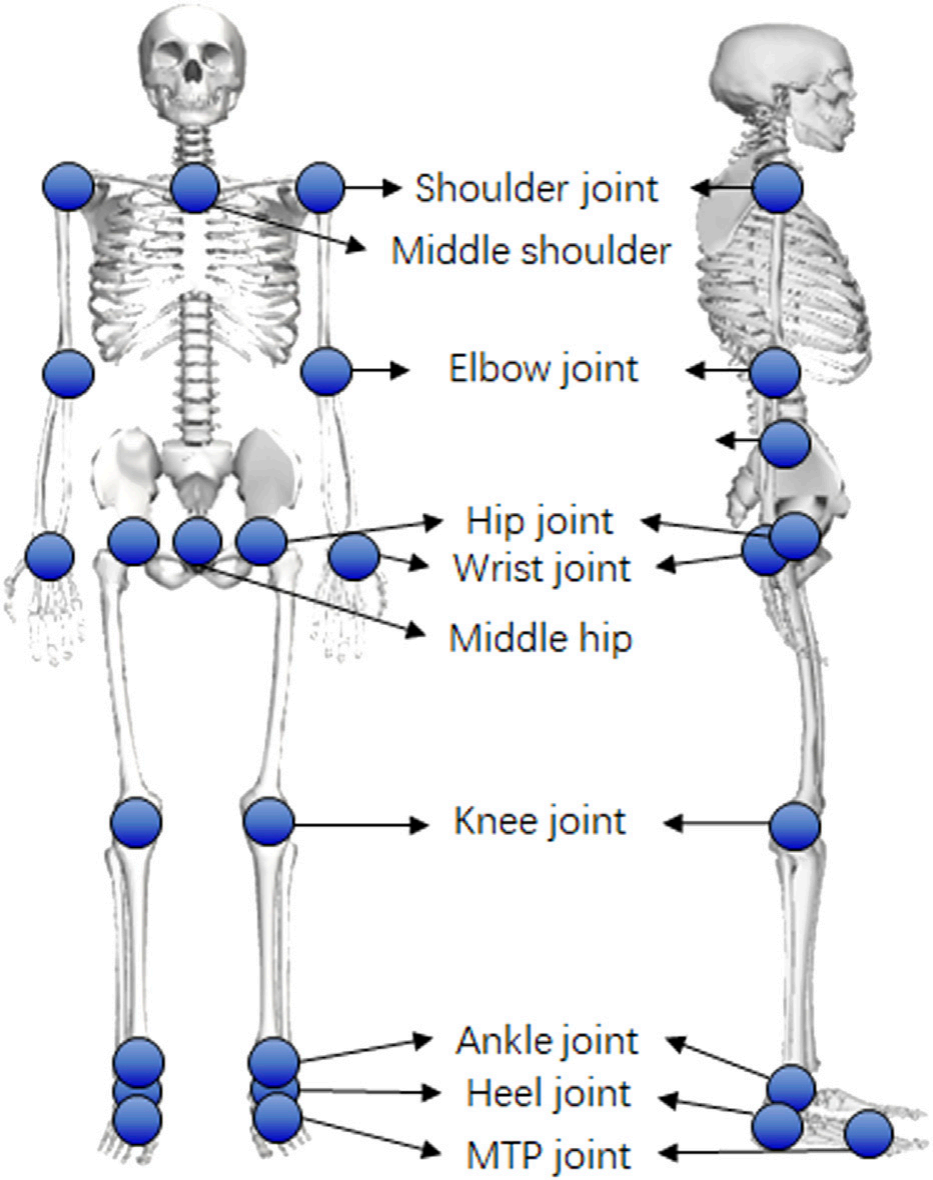
Selected tracking targets in this study.

In addition to traditional biomechanical data, the dataset comprises keypoint trajectories from a markerless motion capture system with varying configurations, including different camera setups and pose estimation algorithms. In this study, the OpenCap dataset included 20 keypoints, including the mid-hip, left and right hips, knees, ankles, heels, small and big toes, mid-shoulder, left and right shoulders, elbows, and wrists. Most of them reflect the joint centers in the OpenSim model (left and right hips, knees, ankles, heels, left and right shoulders, elbows, and wrists) or can be calculated from joint centers (mid-hip and mid-shoulder), except for the small and big toes. Therefore, we manually averaged the trajectories of the big and small toes to estimate the position of the metatarsal joint in the OpenSim model. Consequently, 18 joint centers were targeted for tracking (Figure 2). We selected data from five cameras using the OpenPose high-accuracy setting to form noisy group1 and data from two cameras using the OpenPose default setting to form noisy group2. We also converted the ground truth kinematics into keypoint trajectories as a noise-free group and performed tracking for comparison (Figure 1). Additionally, we averaged the noisy group1 and ground truth data to create a mild noise group. For comparison, trajectories of joint centers with Gaussian noise were additionally created for tracking. In total, five groups of keypoint trajectories with different noise levels (noise-free, mild noise, noisy group1, noisy group2, and Gaussian noise groups) were used for tracking.

For simulations, the OpenSim musculoskeletal model proposed by Uhlrich et al. (2022) employed 33 degrees of freedom (DoFs), including the root joint for the pelvis (6 DoFs, three for rotation and three for translation), bilateral hip (3 DoFs × 2), knee (1 DoF × 2), ankle (1 DoF × 2), subtalar (1 DoF × 2), metatarsophalangeal joint (1 DoF × 2), lumbosacral joint (3 DoFs), shoulder (3 DoFs × 2), and elbow (2 DoFs × 2). The model comprises 80 muscles actuating the lower limbs, along with several ideal torque motors for the lumbosacral joints, and joints from the upper limbs. Six contact spheres were attached to each foot for the simulation of foot‒ground interactions. For each subject, the scaled model provided in the dataset was used for simulation. One movement cycle was sampled for each task. The walking cycle begins when the left foot leaves the ground and ends after a full gait cycle is completed. The squatting cycle begins from a standing position and ends when the person returns to an upright position again.

The mean per joint position error (MPJPE) is a common metric used to evaluate the performance of human pose estimation algorithms. It measures the average distance between the predicted joint positions of a human skeleton and the ground truth joint positions in a given dataset. Smaller MPJPE values indicate better algorithm performance. Recent reports on MPJPE, estimated by pose estimation algorithms using monocular or multiple cameras, range from 16.9 to 45.5 mm (Martinez et al., 2017; Iskakov et al., 2019; Zhu et al., 2022). Compared with the ground truth data, the MPJPE values of the tracked joint centers in this study were 18.4 mm (mild noise group), 37.1 mm (noisy group1), and 37.9 mm (noisy group2) during walking and 15.4 mm, 30.1 mm, and 38.7 mm for squatting, respectively. The trajectories of the joint centers in the Gaussian noise group were established by incorporating Gaussian noise, resulting in an MPJPE of 40.0 mm.

The tracking tasks were treated as optimal control problems, in which the cost function was optimized for minimization. The joint center trajectories from one cycle of walking and squatting with varying noise levels were the primary targets in the cost function (Equation 1). Additional biological terms, including metabolic cost and passive torque terms from the predictive simulation, were adopted to regulate noisy data (Falisse et al., 2019b; Falisse et al., 2022). The cost function ( J ) incorporates multiple terms:

$$J=\int_{0}^{t_{f}}\left({w_{1}E_{traj}^{2}+w}_{2}{\dot{E}}^{2}+w_{3}a^{2}+{w_{4}e_{a}^{2}+w}_{5}u_{a}^{2}+w_{6}T_{p}^{2}\right)dt,$$ (1)

where $t_{f}$ is the gait cycle duration, $E_{traj}$ is the tracking error in trajectories of the joint center, $\dot{E}$ is the metabolic cost, a is the muscle activation, $e_{a}$ is the excitation of the torque motors actuating the joints of the upper limbs, $u_{a}$ is the joint acceleration, $T_{p}$ represents the passive torques, t is the time, and $w_{1-6}$ represents the weight for each term in the cost function based on initial testing during formulation. The weights were set to 10,000; 50; 200; 100,000; 5,000; and 100. $E_{traj}$ was defined as the Euclidean distance of the position of the joint center between the input noise-free and noisy data. By performing forward kinematics, the positions of the joint centers can be obtained, and the distance in the global frame can be subsequently calculated. Since the noisy data may lead to unrealistic joint velocity and acceleration, additional cost terms, including a metabolic penalty term ( $\dot{E}$ ), an activation penalty term ( a ), and an acceleration penalty term ( $u_{a}$ ), were used to generate the physiological behavior of the tracked motion. The metabolic energy rate model proposed by Bhargava et al. (2004) was used for the metabolic penalty term. The passive torque term ( $T_{p}$ ) was used to limit the position of the joint in its reasonable range. As described by Falisse et al. (2019b), optimization involves a set of constraints, including muscle activation dynamics, muscle contraction dynamics, torque activation dynamics, skeleton dynamics, zero pelvis residual forces, and state continuity. To prevent self-collision, we impose constraints on the distance between joint centers, ensuring that the lower limbs do not intersect. We also implemented cyclic constraints in the walking task to increase the convergence speed.

Since the metabolic energy rate term was incorporated for noise regulation, the inclusion of muscle–tendon units (MTUs) within the simulations is necessary. In line with Falisse et al. (2022), muscle excitation–activation coupling was modeled using Raasch’s model (Raasch et al., 1997; De Groote et al., 2009), whereas a Hill-type model described muscle–tendon interactions (Zajac, 1989; De Groote et al., 2016). The MTU parameters were extracted from the scaled models. Skeletal motion was depicted through Newtonian rigid body dynamics using compliant Hunt–Crossley foot–ground contacts with a stiffness set at 1 MPa and contact spheres with radii set at 0.032 m. Other parameters in the contact model, including dissipation, friction coefficients, and transition velocity, were set up as described by Falisse et al. (2019b). The ideal torque motor dynamics were represented via a linear first-order approximation. The muscle–tendon lengths, velocities, and moment arms were fitted as polynomial functions of the joint positions and velocities to improve the computational efficiency. The state variables included the joint position, velocity of all DoFs, normalized tendon force, and activation of the muscle and torque actuators. The control variables included derivatives of muscle activations and excitation of the torque actuator. Other control variables included joint acceleration and the tendon force derivative. Inverse kinematics was performed based on the tracked joint center trajectories of each group to establish initial guesses for joint positions, velocities, and accelerations. The initial guesses for other variables and the bounds of the variables were set up, and all the design variables were scaled, as described by Falisse et al. (2019b).

OpenSim 4.3 was used for musculoskeletal modeling (Seth et al., 2018), whereas OpenSimAD provided automatic differentiation (Falisse et al., 2019a), generating necessary derivatives for evaluating forward kinematics and inverse dynamics functions. CasADi was used to formulate the optimization problem, and IPOPT was used as an optimization solver in the direct collocation scheme with the Radau quadrature (Andersson et al., 2019). Parallel formulation was used to ensure efficient execution in CasADi. Based on preliminary findings, the tasks were discretized into 30 mesh intervals to balance the optimization speed and accuracy.

Sensitivity studies on metabolic weightings (0 (M0) and 10 times the default weighting (M10)) and passive torque weightings (0 (P0) and 10 times the default weighting (P10)) were performed for noisy group2. Additionally, we performed sensitivity studies on the number of mesh intervals, in which 40 mesh intervals (N40) and 50 mesh intervals (N50) were applied for the noisy group2 data. In total, we performed 220 optimization cases, including both walking and squatting tracking tasks, across five data groups (noise-free, mild noise, noisy group1, noisy group2, and Gaussian noise group) and six additional sensitivity groups (M0, M10, N40, N50, P0, and P10) for each task, involving 10 subjects. For comparison, we used the OpenCap pipeline with default settings to track the inverse kinematics data derived from the augmented markers of the two activities in the noisy group1 and 2 data. The ground truth kinematics derived from the optical motion capture of the two activities were also tracked using OpenCap with default settings for comparison.

After the optimization process was finished, the simulated kinematics, joint moments, and GRFs were generated. To account for subject variability, we normalized joint moments and GRFs by total body mass. The data were compared with the ground truth kinematics (derived from optical motion capture), joint moments, and GRF data. The mean absolute errors (MAEs) were calculated and compared between each group of data to show the performance in tracking and estimating kinetics. The kinematics derived from the augmented markers in the OpenCap dataset were also analyzed for comparison.

## Results

**FIGURE 3:**
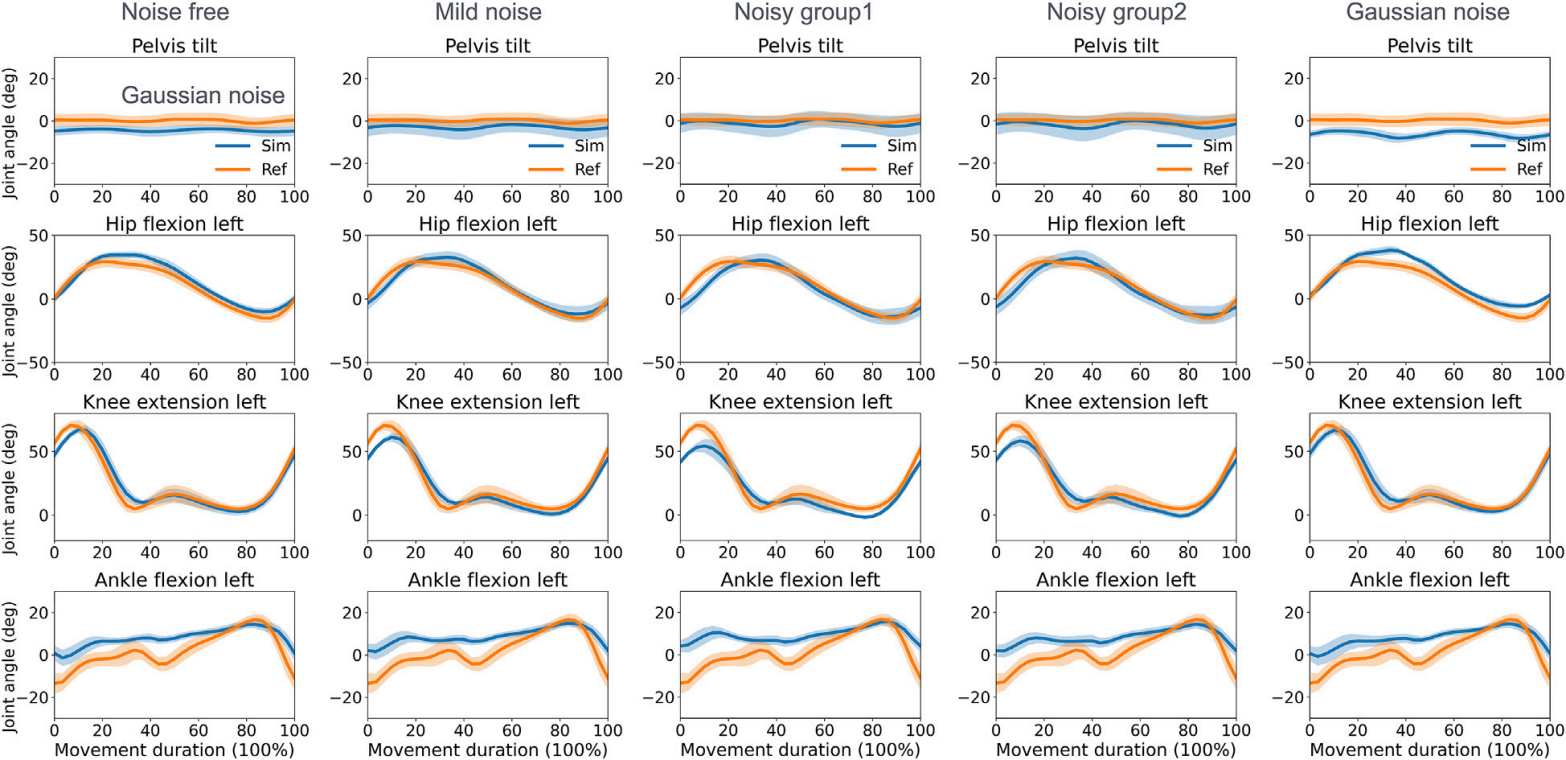
Reference and tracked kinematics using the direct collocation method (mean and standard deviation) in the walking task. Ref, ground truth data derived from optical motion capture; Sim, simulation results.

**FIGURE 4:**
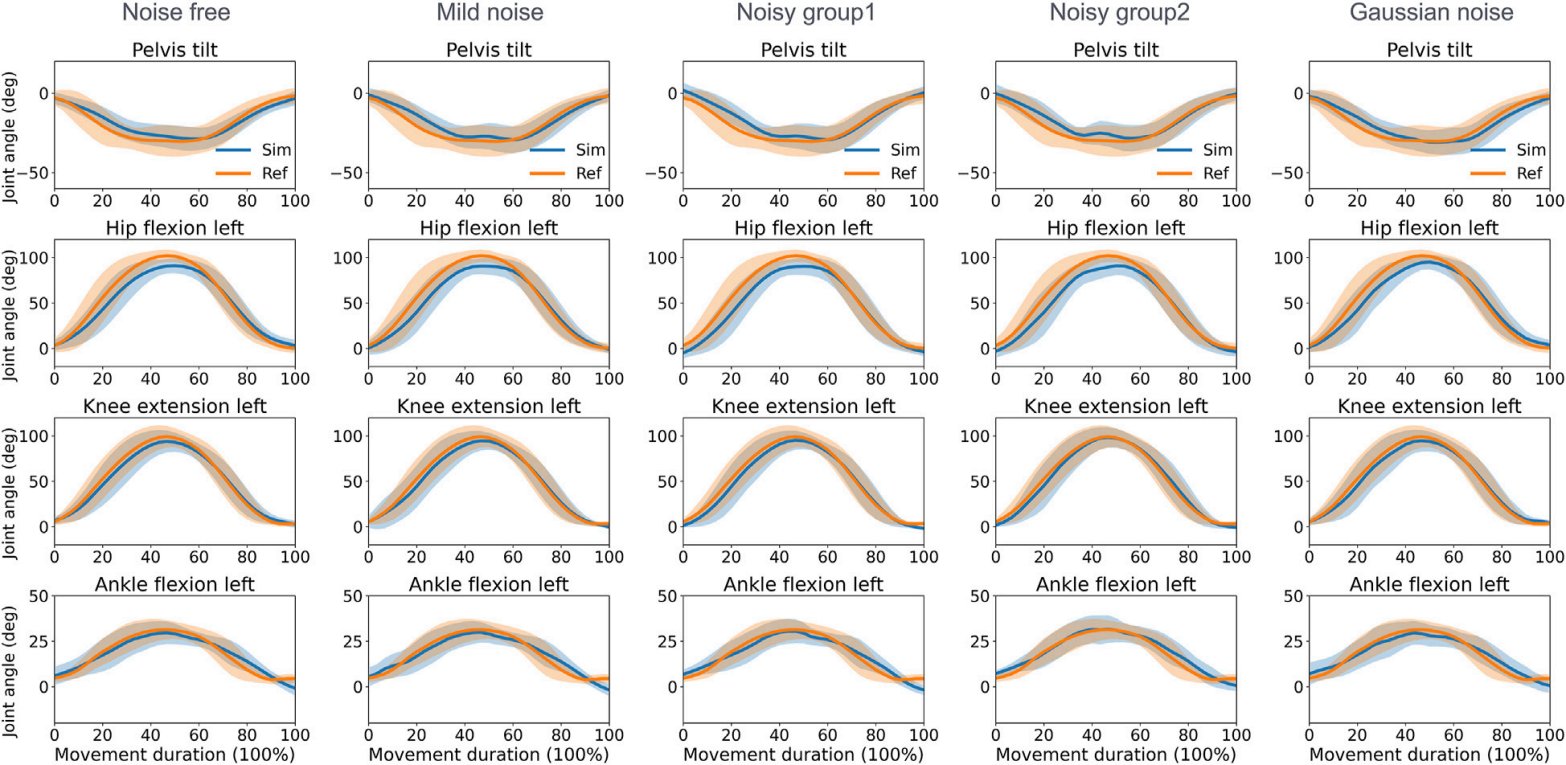
Reference and tracked kinematics using the direct collocation method (mean and standard deviation) in the squatting task. Ref, ground truth data derived from optical motion capture; Sim, simulation results.

The direct collocation method successfully completed all tracking tasks (Figures 3, 4). As shown in Table 1, the MAEs for each activity were averaged across movements and participants. In the walking task, the MAEs were 4.2° for the noise-free group, 5.1° for the mild noise group, 7.0° for noisy group1, 6.1° for noisy group2, and 4.6° for the Gaussian noise group for knee flexion angles across 10 participants. As the noise level increased, the accuracy of hip joint tracking decreased, with MAEs for hip flexion angles of 4.7°, 4.5°, 5.6°, 6.6°, and 6.8° in the corresponding groups. The ankle flexion angles revealed MAEs of 7.1°, 7.5°, 8.1°, 7.5°, and 7.1° in the respective groups. During the squatting task, the mean hip flexion angles had MAEs of 8.2°, 8.3°, 8.8°, 9.0°, and 8.0°, whereas knee flexion angles recorded 5.1°, 4.9°, 5.3°, 5.1°, and 4.8° in the respective groups. Ankle flexion angles showed MAEs of 3.7°, 3.7°, 3.4°, 3.5°, and 4.1° in the respective groups. Additional kinematics and error data for other lower limbs are detailed in Supplementary Material 1, 2.

**TABLE 1:** Mean absolute error of kinematics.

Noise level	Method	Walking		Squatting	
		Rotations (°)	Translations (mm)	Rotations (°)	Translations (mm)
Noise-free	This study	4.7 (2.7–7.4)	12.2 (5.3–16.8)	5.4 (1.2–12.0)	15.5 (3.2–27.0)
	OpenCap (Tracking)	1.0 (0–2.9)	8.0 (1.0–12.5)	1.9 (0–5.7)	8.6 (0.4–17.3)
Mild noise	This study	4.9 (2.6–7.8)	11.0 (6.1–14.4)	5.5 (1.4–12.9)	17.6 (6.8–26.6)
Noisy group1	This study	5.7 (3.2–8.3)	11.9 (8.8–13.8)	5.6 (1.5–12.7)	19.8 (10.8–25.6)
	OpenCap (IK)	3.9 (1.4–7.6)	12.0 (8.2–16.3)	3.4 (1.3–6.8)	13.4 (10.2–15.1)
	OpenCap (Tracking)	5.0 (2.2–9.5)	21.8 (8.6–40.1)	3.9 (1.4–8.1)	18.2 (10.2–25.9)
Noisy group2	This study	6.0 (3.5–8.1)	13.4 (10.4–15.2)	5.6 (1.7–13.0)	20.2 (13.0–23.9)
	This study (M0)	5.7 (3.2–8.0)	12.4 (11.0–14.1)	5.3 (1.6–11.3)	18.6 (12.7, 23.7)
	This study (M10)	8.1 (5.1–11.1)	15.2 (11.1–20.3)	7.4 (1.6–16.8)	30.0 (12.8, 52.9)
	This study (N40)	5.9 (3.4–8.1)	13.3 (10.4–15.0)	5.5 (1.7–12.4)	19.2 (13.0, 23.1)
	This study (N50)	5.9 (3.4–8.1)	13.5 (10.5–15.4)	5.5 (1.6–12.3)	18.6 (12.8, 22.9)
	This study (P0)	7.8 (5.5–13.2)	17.2 (13.9–21.6)	8.1 (2.6–18.4)	23.5 (14.3–36.5)
	This study (P10)	5.8 (3.3–8.3)	12.9 (9.7–14.6)	6.5 (1.4–12.4)	22.1 (13.1–32.1)
	OpenCap (IK)	4.4 (2.2–7.1)	12.7 (12.0–13.8)	3.9 (1.6–7.0)	16.3 (14.2–20.1)
	OpenCap (Tracking)	4.5 (2.3–7.1)	22.0 (12.3–40.5)	4.4 (1.7–9.5)	19.6 (13.7–24.0)
Gaussian noise	This study	5.3 (2.5–8.8)	13.2 (6.3–18.7)	5.7 (1.8–11.2)	15.9 (7.1–24.0)

Note: Errors for each activity were averaged over participants and reported as an average over movements and degrees of freedom (rotations: three for pelvis orientation, three for the lumbar, three per hip, one per knee, and two per ankle; translations: three for the pelvis position). Kinematic errors are presented as the mean and range over the degrees of freedom. M0, with a metabolic weight of 0; M10, with 10 times the default metabolic weight; P0, with a passive torque weight of 0; P10, with 10 times the default passive torque weight. The default number of mesh intervals is 30. N40, with 40 mesh intervals; N50, with 50 mesh intervals. IK, inverse kinematics data from trajectories of augmented markers in the OpenCap dataset. Tracking simulated kinematics by tracking the IK, data using OpenCap default settings.

**FIGURE 5:**
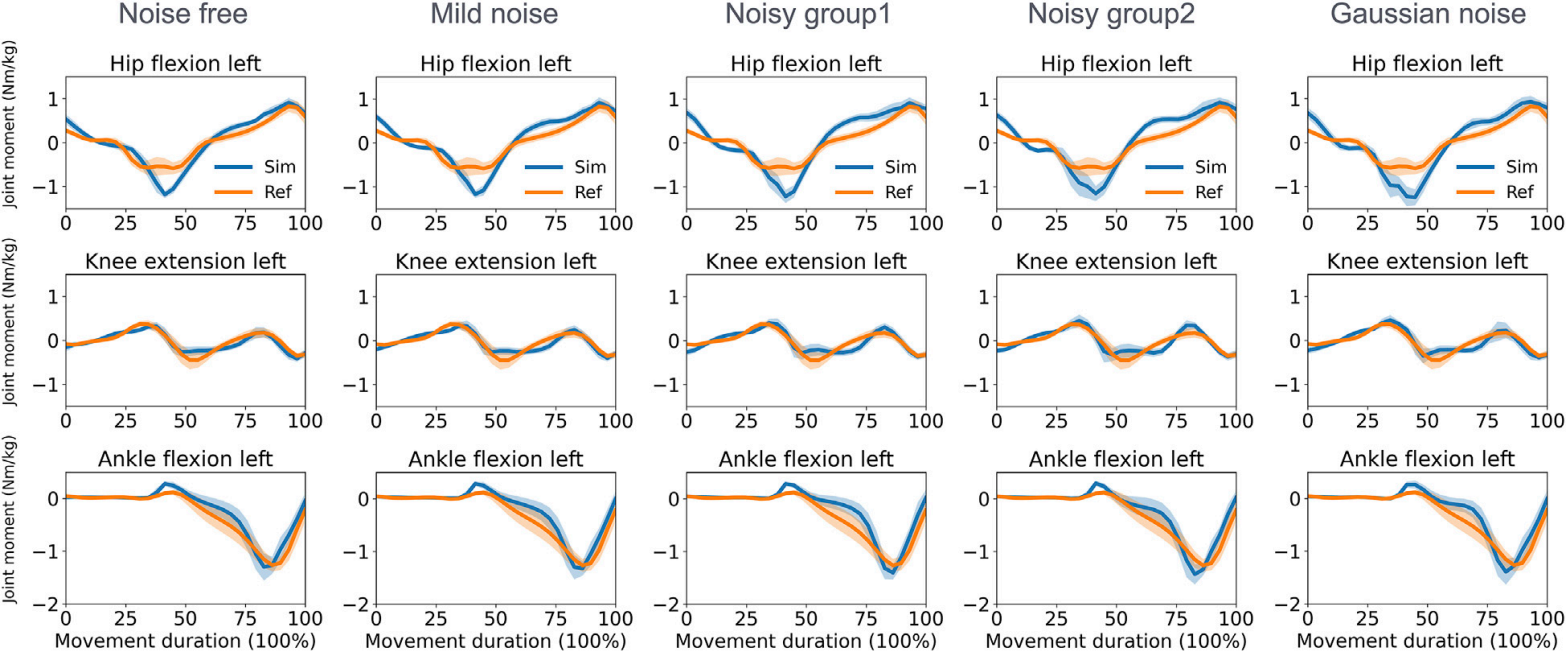
Normalized joint moment (mean and standard deviation) estimated by the direct collocation method and the reference data in the walking task. Ref, ground truth data derived from inverse dynamics based on optical motion capture and force plate data; Sim, simulation results.

**FIGURE 6:**
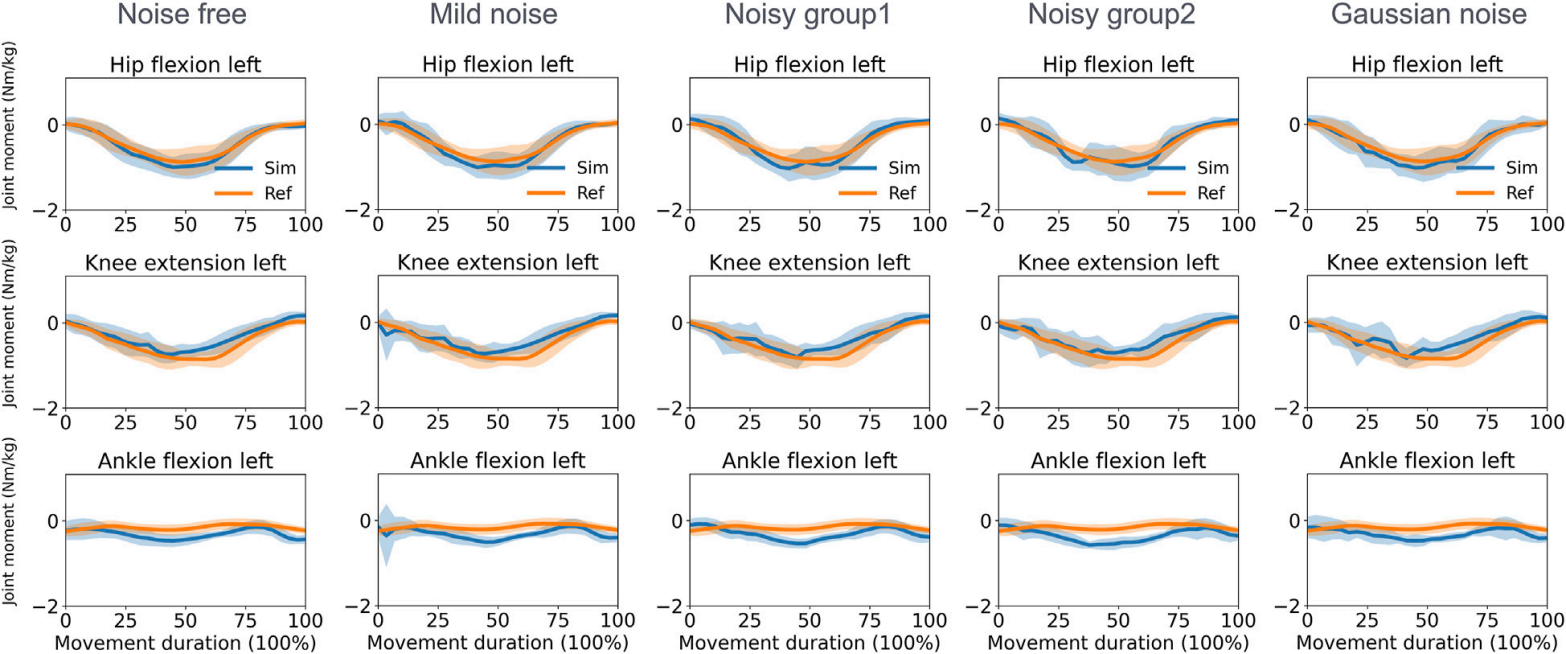
Normalized joint moment (mean and standard deviation) estimated by the direct collocation method and the reference data in the squatting task. Ref, ground truth data derived from inverse dynamics based on optical motion capture and force plate data; Sim, simulation results.

For joint moment estimation during the walking task (Figure 5), the knee and ankle joints achieved higher accuracy than hip joints when assessed against the ground truth. Specifically, the MAEs for the knee flexion moment were 0.103, 0.113, 0.127, 0.129, and 0.116 Nm/kg in the noise-free, mild noise, noisy group1, noisy group2, and the Gaussian noise group, respectively. For the ankle flexion moment, the corresponding MAEs were 0.130, 0.133, 0.145, 0.131, and 0.138 Nm/kg. The hip flexion moment had MAEs of 0.182, 0.204, 0.242, 0.246, and 0.249 Nm/kg in the respective groups. During squatting (Figure 6), the MAEs of the ankle flexion moments were 0.207, 0.219, 0.217, 0.253, and 0.227 Nm/kg in the noise-free, mild noise, noisy group1, noisy group2, and the Gaussian noise group, respectively. The MAEs of the knee flexion moments were 0.177, 0.196, 0.198, 0.197, and 0.221 Nm/kg, whereas the mean MAEs of the hip flexion moments were 0.125, 0.135, 0.141, 0.161, and 0.178 Nm/kg in the respective groups. Table 2 lists the MAEs of the joint moment for each activity. The joint moments and errors of the other lower limbs are reported in Supplementary Material 3, 4.

**TABLE 2:** Normalized mean absolute error of joint moment.

Noise level	Method	Walking	Squatting
		All degrees of freedom (Nm/kg)	All degrees of freedom (Nm/kg)
Noise-free	This study	0.10 (0.03–0.19)	0.18 (0.01–0.47)
	OpenCap	0.10 (0.02–0.20)	0.17 (0.01–0.35)
Mild noise	This study	0.11 (0.03–0.21)	0.18 (0.02–0.42)
Noisy group1	This study	0.13 (0.03–0.25)	0.18 (0.03–0.41)
	OpenCap	0.20 (0.05–0.36)	0.18 (0.02–0.37)
Noisy group2	This study	0.14 (0.04–0.28)	0.18 (0.04–0.39)
	This study (M0)	0.20 (0.06–0.45)	0.22 (0.08–0.47)
	This study (M10)	0.15 (0.04–0.31)	0.17 (0.03–0.32)
	This study (N40)	0.14 (0.04–0.28)	0.18 (0.05–0.37)
	This study (N50)	0.14 (0.04–0.27)	0.18 (0.05–0.39)
	This study (P0)	0.15 (0.04–0.33)	0.18 (0.06–0.39)
	This study (P10)	0.15 (0.04–0.31)	0.21 (0.05–0.45)
	OpenCap	0.19 (0.05–0.37)	0.19 (0.03–0.41)
Gaussian noise	This study	0.13 (0.04–0.25)	0.18 (0.07–0.35)

Note: Errors for each activity were averaged over all participants (n = 10) and are reported as an average over movements and degrees of freedom (three for lumbar, three per hip, one per knee, and two per ankle). Kinetic errors are presented as the mean and range across degrees of freedom.

**FIGURE 7:**
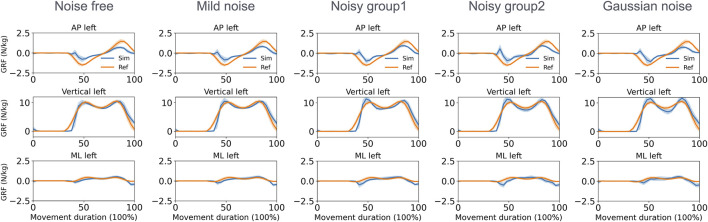
Normalized GRF of the left foot estimated by the direct collocation method and the reference data of the walking task (AP, anterior–posterior; ML, medial–lateral).

**FIGURE 8:**
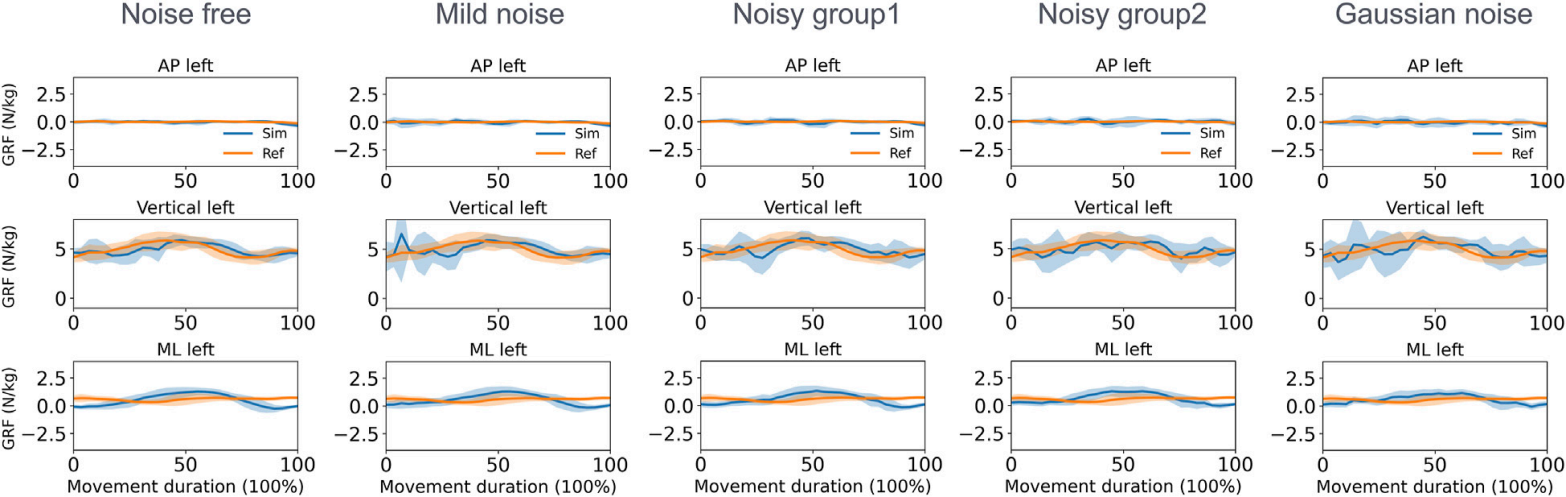
Normalized GRF of the left foot estimated by the direct collocation method and the reference data of the squatting task (AP, anterior–posterior; ML, medial–lateral).

With respect to the GRF estimation in the walking task (Figure 7), the mean MAEs for both feet in the vertical direction varied from 0.74 to 0.95 N/kg across the five groups. During squatting (Figure 8), these MAEs ranged from 0.80 to 1.25 N/kg (Table 3). Additional GRF data and error information for each group are available in Supplementary Material 5, 6.

**TABLE 3:** Normalized mean absolute error of ground reaction force (N/kg).

Noise level	Method	Walking			Squatting		
		AP	Vertical	ML	AP	Vertical	ML
Noise-free	This study	0.36	0.74	0.12	0.16	0.80	0.63
	OpenCap	0.24	0.71	0.11	0.11	0.36	0.50
Mild noise	This study	0.34	0.77	0.13	0.21	0.95	0.58
Noisy group1	This study	0.33	0.84	0.15	0.20	1.01	0.57
	OpenCap	0.43	1.11	0.23	0.10	0.40	0.51
Noisy group2	This study	0.35	0.87	0.18	0.24	1.07	0.57
	This study (M0)	0.56	1.59	0.24	0.30	1.56	0.68
	This study (M10)	0.33	0.80	0.20	0.19	0.81	0.43
	This study (N40)	0.35	0.85	0.19	0.25	1.09	0.55
	This study (N50)	0.35	0.84	0.18	0.26	1.02	0.56
	This study (P0)	0.29	1.13	0.20	0.25	1.15	0.54
	This study (P10)	0.39	0.84	0.20	0.22	1.21	0.66
	OpenCap	0.46	1.63	0.21	0.14	0.59	0.50
Gaussian noise	This study	0.35	0.95	0.16	0.28	1.25	0.52

Note: Errors for each activity were averaged over all the participants (n = 10), and the reported mean is an average over movements and feet.

## Discussion

This study performed a sensitivity analysis to explore the capabilities of the direct collocation method for kinetics estimation based on keypoint trajectories detected from pose estimation algorithms. Walking and squatting were selected as the tracking activities. To illustrate the effect of errors inherent in pose estimation algorithms, various noise levels of trajectories of joint centers were used for tracking. Our results indicate that the direct collocation method robustly tracks movements despite noise. Furthermore, our study highlights the feasibility and practical considerations of applying the direct collocation method in conjunction with markerless motion capture for biomechanical analysis.

A key finding was that the direct collocation approach, which incorporates both tracking and biological terms in the cost function, could robustly track noisy joint center trajectories and estimate joint moments and GRFs. The tracking errors remained consistent across various noise scenarios, largely because of the biological terms included in the cost function. The incorporation of constraints in the optimization process—such as state continuity, muscle activation dynamics, contact dynamics, and collision avoidance—ensures realistic and plausible model movements. Consequently, the method was robust to typical noise levels encountered with markerless motion capture systems, with kinematics converging into recognizable patterns, which is consistent with findings from previous studies by Falisse et al. (2019b) and Falisse et al. (2022). In addition to previous studies simulating walking, our study revealed that a tracking term in combination with biological terms could also be used to simulate squatting activity. Unlike economical movements such as walking, squatting is an energy-consuming activity. The robust performance in the squatting task indicates that the current settings in the collocation scheme are also suitable for tracking noneconomical activities.

The inclusion of a metabolic energy rate term played a significant role in managing noisy data and achieving biologically realistic kinetic estimations. Although excluding this term (in the M0 setting for noisy group2) slightly improved tracking accuracy, it resulted in higher errors for joint moment and GRF estimations than the default settings. This study adopted the metabolic energy rate model proposed by Bhargava et al. (2004), in which muscle excitation, activation, muscle fiber kinematics, and fiber-related forces were included to estimate the metabolic energy rate. This resulted in smooth and physiologically sound joint moments. In contrast, the OpenCap pipeline used joint position, velocity, and acceleration as tracking terms in the cost function (Uhlrich et al., 2023). This setting has certain benefits in estimating the joint moment when the input data are noise-free. However, the tracked joint acceleration, as the second derivative of the joint position, is susceptible to inaccuracies in the joint position, which can lead to deviations in joint acceleration and subsequently influence the results of the joint moment estimation. This could explain why, despite the OpenCap pipeline providing more accurate kinematics from augmented markers, the errors in joint moment estimation remained at the same levels as those observed in our study. In contrast, this study, which includes a metabolic energy rate term, could robustly estimate joint moments and GRFs against different noise levels. However, excessive metabolic weighting (M10) led to deviations from reference data, indicating a need for a balanced setting of the metabolic term for optimal performance. For example, low metabolic weighting could be assigned to energy-consuming activities.

Despite robust tracking and joint moment estimation, certain challenges persist. For example, the ankle flexion angle in the walking task had a smaller range of motion than the reference data. This issue also occurred in predictive simulations (Falisse et al., 2019b; Falisse et al., 2022; D'Hondt et al., 2024), indicating that current modeling of foot- or energy-utilizing strategies may deviate from that of humans. Interestingly, the group with zero metabolic weighting (M0) performed better in tracking ankle flexion movements (see Supplementary Material 1). This result suggests that metabolic weighting may be divided among specific muscle groups and that low metabolic weighting could be applied to energy-consuming activities involving those muscle groups. Additionally, discrepancies between the pelvis tilt and hip flexion angles emerged, where incorrect pelvis tilt estimations impacted the accuracy of hip flexion. This is because the orientation of the pelvis cannot be easily tracked by the positions of the hip joint centers. Since the pelvis is the root segment of the human model, the hip flexion angle may also be influenced. This issue is prevalent in pose estimation algorithms and remains difficult to resolve, even with multiple cameras (Wren et al., 2023). To address this problem, incorporating an additional inertial measurement unit (IMU) attached to the pelvis may be a suitable way to obtain precise orientation data.

Although our GRF profiles approximated the ground truth data, minor discrepancies arose in the walking task—specifically, in the initial contact time and magnitude. Several reasons may cause this issue. First, the thickness and material properties of the subject’s feet and shoes were not precisely modeled because of missing information in the ground truth data. Furthermore, the lumbar and knee joints were treated as joints with only rotational DoFs. The omission of their cushioning effects in the translational DoFs during simulation may contribute to GRF estimation errors. To alleviate this issue, calibrating the stiffness of the contact element and adjusting the height of the floor in the simulation scheme may improve the results (Serrancoli et al., 2019). Increasing the number of contact elements may also enhance the performance.

The estimation of joint moments in our study yielded error levels comparable to those from the OpenCap pipeline, despite fundamental differences in the cost function. Notably, OpenCap employs distinct weights to track various joint kinematics and activities, along with additional settings such as heel contact constraints for specific tasks. In contrast, our method applied uniform weights across all joint centers and used the same settings for both activities, except for incorporating cyclic constraints for walking. Consequently, while there was a compromise in kinematics tracking accuracy, our method maintained robust kinetics estimation. It is expected that introducing joint-specific and activity-specific weighting could further enhance the performance.

In both the walking and squatting tasks, the direct collocation method consistently provided more accurate estimations for the ankle and knee joint moments than for the hip joints. Several factors might contribute to this tendency. First, the inaccuracy in tracking hip joint angles, as mentioned earlier, plays a significant role. Additionally, joint moments are heavily influenced by the GRF, and its accuracy in the direct collocation scheme heavily relies on input kinematics and the model. In this study, the trunk was modeled as a single rigid body, a simplification that might result in a loss of detail regarding kinematics, inertia, and mass distribution, thereby leading to greater deviations in joint moment estimations adjacent to the trunk. On the other hand, the ground truth joint moments were calculated using OpenSim’s inverse dynamics function, which permitted certain pelvis residual torques to actuate degrees of freedom. However, the current direct collocation scheme constrained these residual forces to 0. Therefore, a detailed trunk model may be helpful in improving the quality of kinetics estimation.

This study has several limitations. Compared with previous methods, this study yielded greater tracking errors in joint angles when noise-free kinematics were tracked (Lin and Pandy, 2017; Uhlrich et al., 2023). The main reason is that to maintain robust tracking performance across different noise levels, our study incorporated biological terms for regulating noisy joint center trajectories. In our additional sensitivity studies, removing the metabolic term (M0) improved tracking accuracy, whereas its inclusion in the M10 configuration led to decreased accuracy. In terms of kinetics estimation, M0 generated more errors in the joint moment estimation than the default setting did in this study. Although the biological terms can constrain the noise trajectories into physiological movements and joint moments, they come at the expense of tracking performance. Furthermore, the selected joint centers might be insufficient for accurate joint angle tracking. Therefore, future studies could explore more generalized cost functions to strike a balance between robustness and accuracy. To further improve the robustness of joint moment estimation, the weight for each term in the cost function can be further divided and set up according to the input data quality and prior knowledge of the activities. Additionally, variations in the estimated joint moment errors across subjects and joints indicate that this method is currently better suited to reflect general activity dynamics than subject-specific dynamics when similar movements are performed.

## Conclusion

The direct collocation method, which incorporates both tracking and biological terms into the cost function, can robustly estimate joint moments during walking and squatting across various noise levels. Future studies should aim to develop more comprehensive cost functions to achieve the optimal balance between robustness and accuracy.

## Data Availability

The original contributions presented in the study are included in the article/Supplementary Material; further inquiries can be directed to the corresponding authors.
