# Supplementary material for: Robust kinetics estimation from kinematics via direct collocation
Source: Front Bioeng Biotechnol. 2024 Dec 18;12:1483225. doi: 10.3389/fbioe.2024.1483225 (PMC11688375; doi:10.3389/fbioe.2024.1483225)
Supplement: Supplementary file 4 [file DataSheet5.docx]

Supplementary material 5


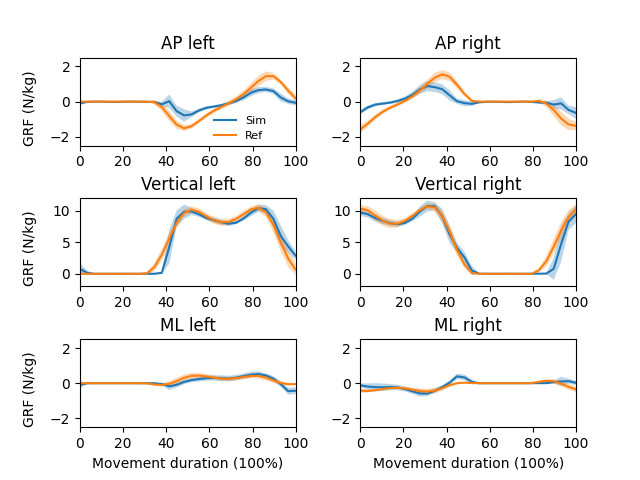


Fig S5-1. Normalized ground reaction force (GRF) of the reference data in the walking task and results of direct collocation method (Noise-free level with default setting)


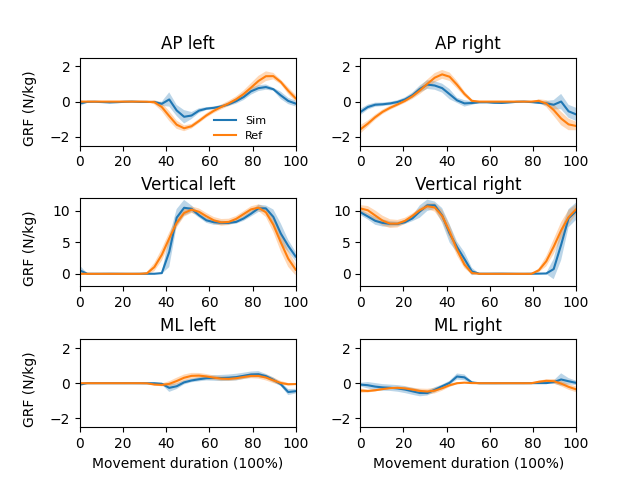


Fig S5-2. Normalized ground reaction force (GRF) of the reference data in the walking task and results of direct collocation method (Mild noise level with default setting)  
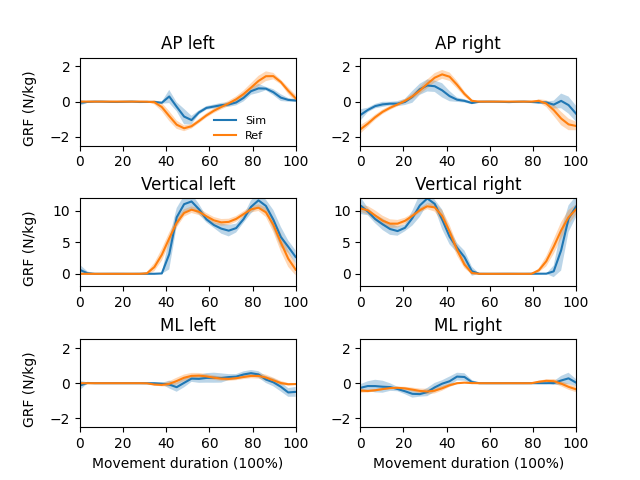


Fig S5-3. Normalized ground reaction force (GRF) of the reference data in the walking task and results of direct collocation method (Gaussian noise with default setting)


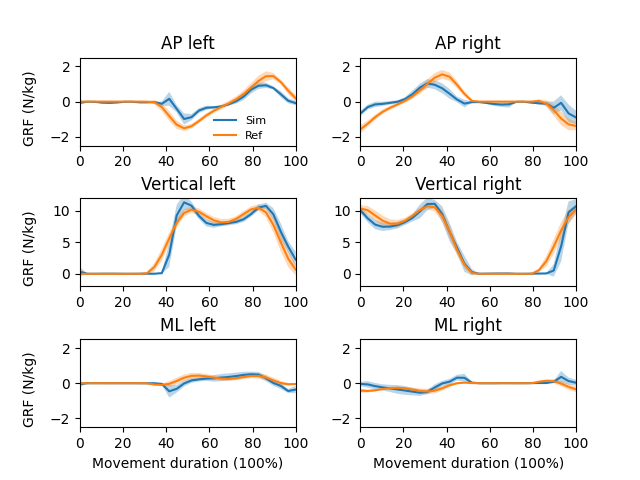


Fig S5-4. Normalized ground reaction force (GRF) of the reference data in the walking task and results of direct collocation method (Noisy group1 level with default setting)


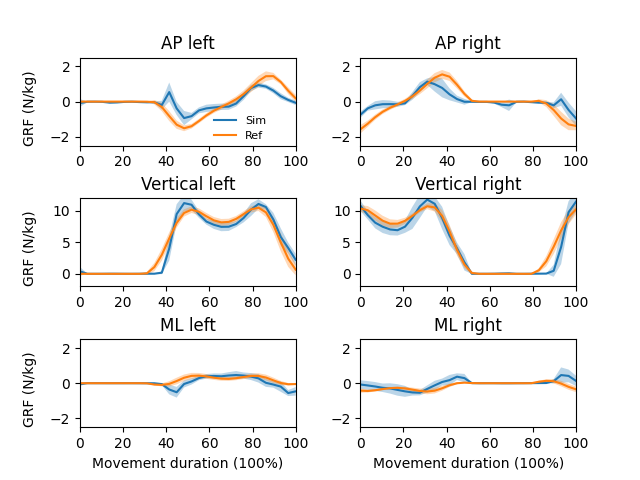


Fig S5-5. Normalized ground reaction force (GRF) of the reference data in the walking task and results of direct collocation method (Noisy group2 level with default setting)


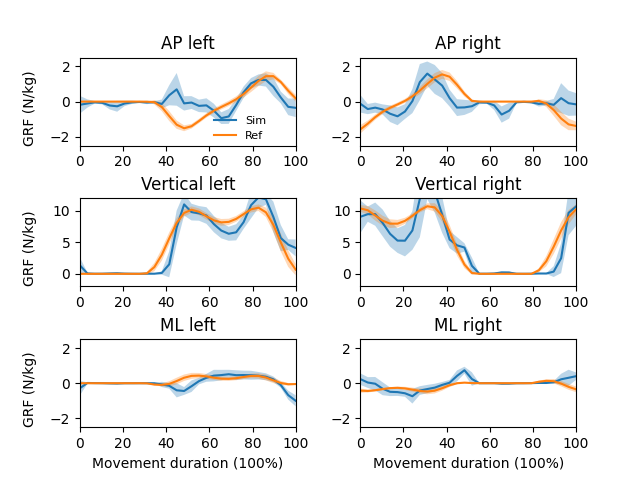


Fig S5-6. Normalized ground reaction force (GRF) of the reference data in the walking task and results of direct collocation method (Noisy group2 level with zero metabolic weighting (M0))


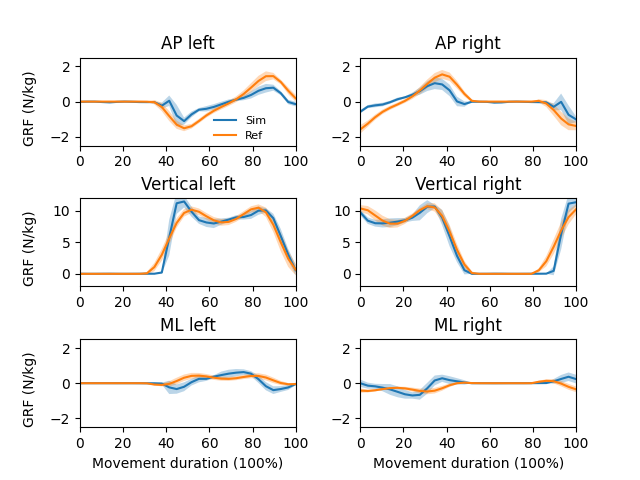


Fig S5-7. Normalized ground reaction force (GRF) of the reference data in the walking task and results of direct collocation method (Noisy group2 level with ten times of metabolic weighting (M10))


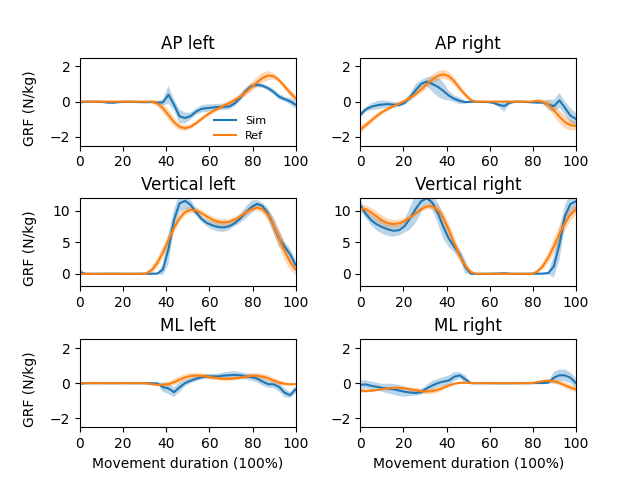


Fig S5-8. Normalized ground reaction force (GRF) of the reference data in the walking task and results of direct collocation method (Noisy group2 level with 40 mesh intervals (N40))


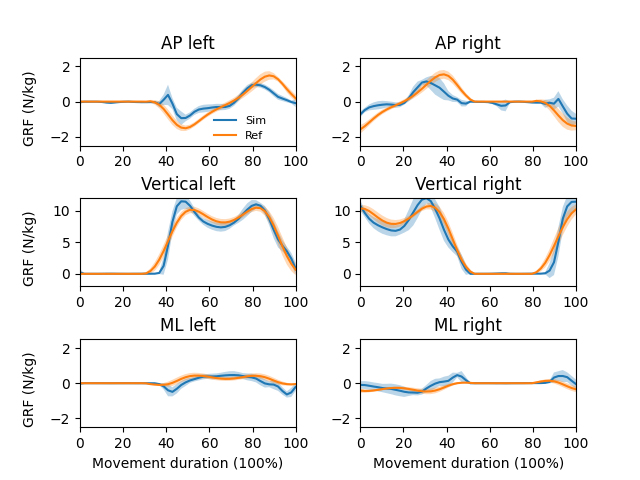


Fig S5-9. Normalized ground reaction force (GRF) of the reference data in the walking task and results of direct collocation method (Noisy group2 level with 50 mesh intervals (N50))   
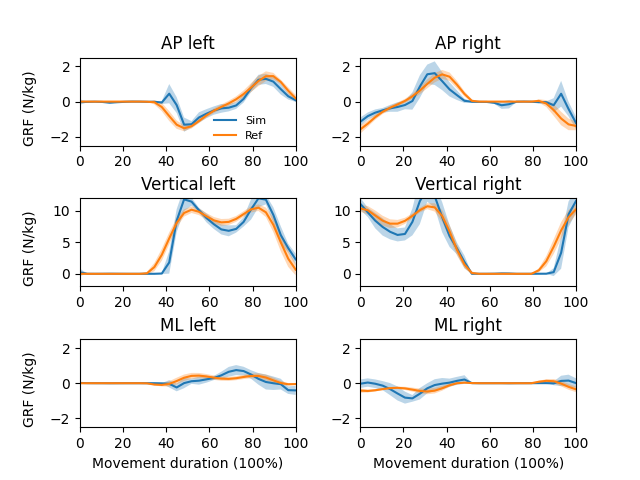


Fig S5-10. Normalized ground reaction force (GRF) of the reference data in the walking task and results of direct collocation method (Noisy group2 level with zero passive torque weighting (P0))   
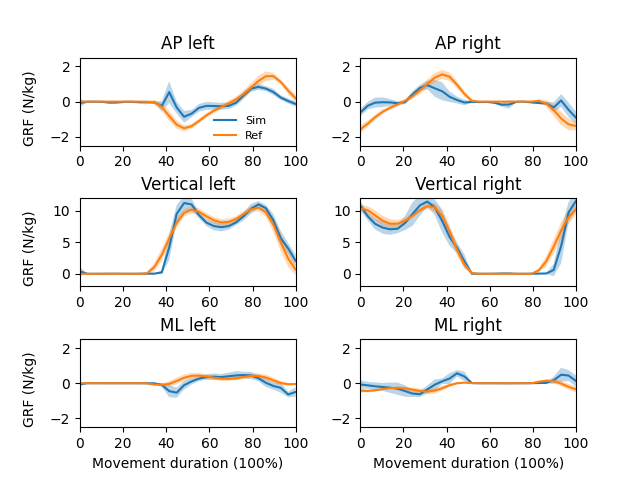


Fig S5-11. Normalized ground reaction force (GRF) of the reference data in the walking task and results of direct collocation method (Noisy group2 level with ten times of passive torque weighting (P10))


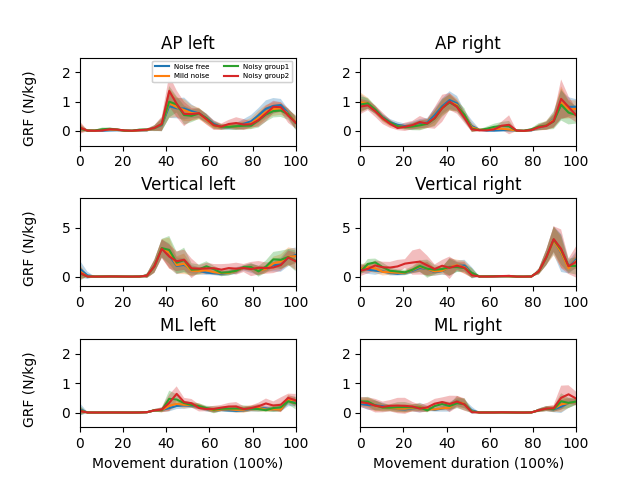


Fig S5-12. Mean absolute errors and standard deviations of normalized ground reaction force (GRF) between the reference data and results of direct collocation method
