# Supplementary material for: Robust kinetics estimation from kinematics via direct collocation
Source: Front Bioeng Biotechnol. 2024 Dec 18;12:1483225. doi: 10.3389/fbioe.2024.1483225 (PMC11688375; doi:10.3389/fbioe.2024.1483225)
Supplement: Supplementary file 5 [file DataSheet2.docx]

Supplemental material 2


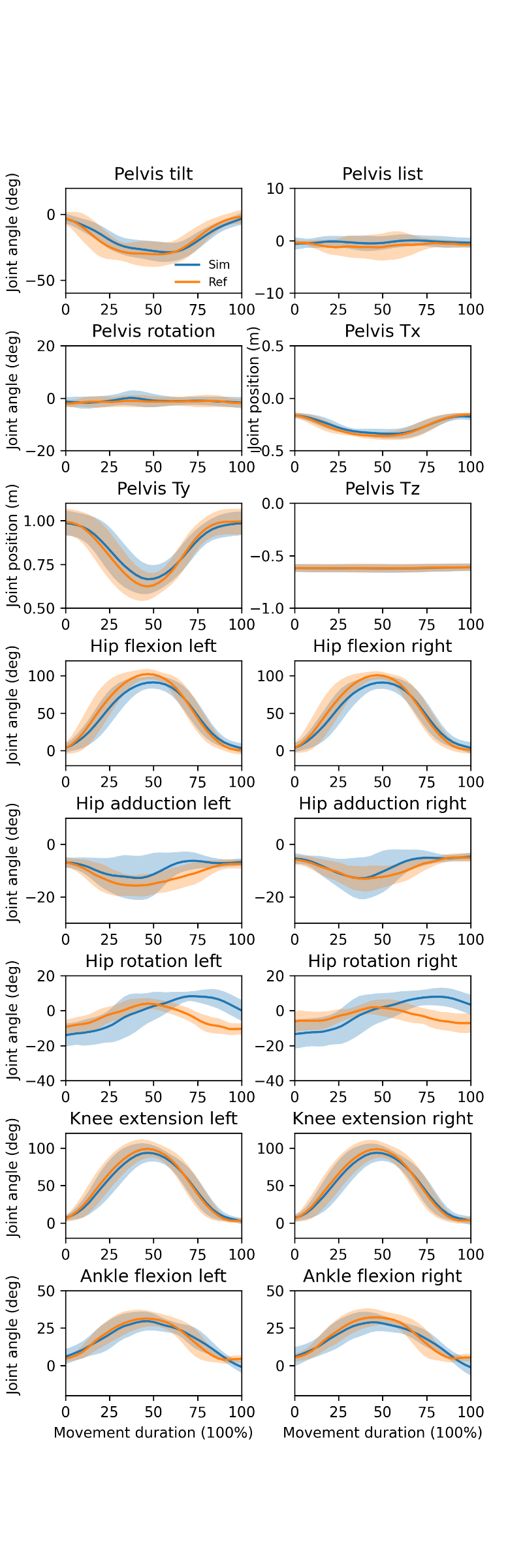


Fig S2-1. Reference kinematics in the squatting task and kinematics tracked using direct collocation method (Noise free level with default setting)


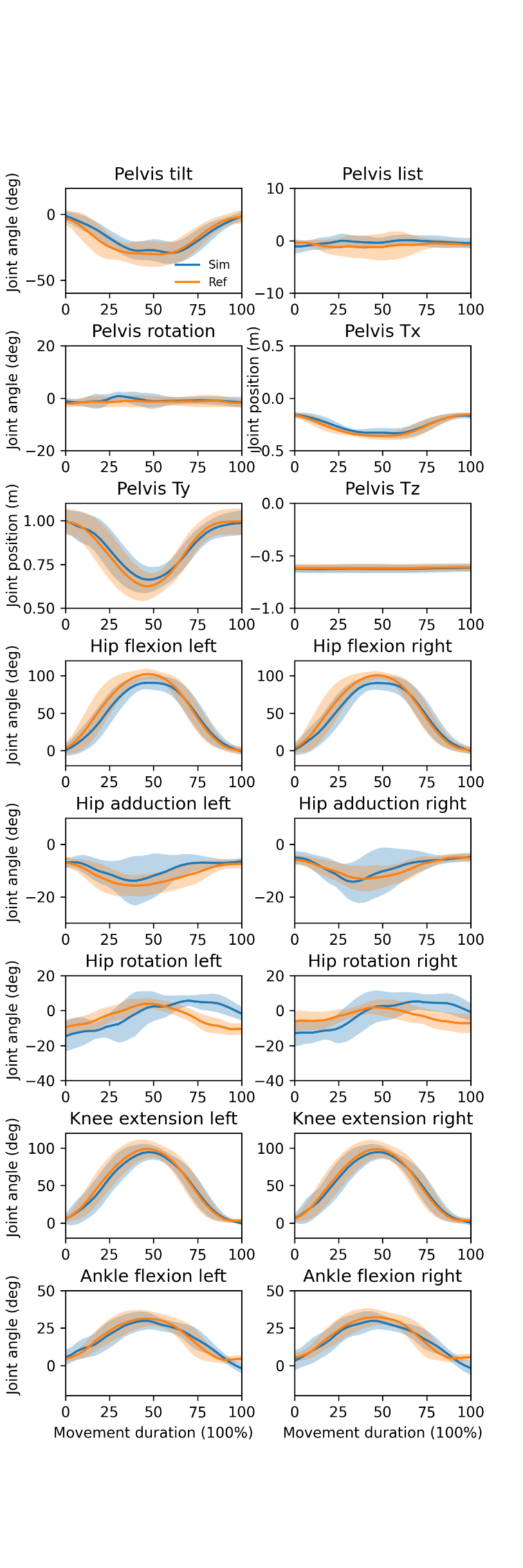


Fig S2-2. Reference kinematics in the squatting task and kinematics tracked using direct collocation method (Mild noise level with default setting)


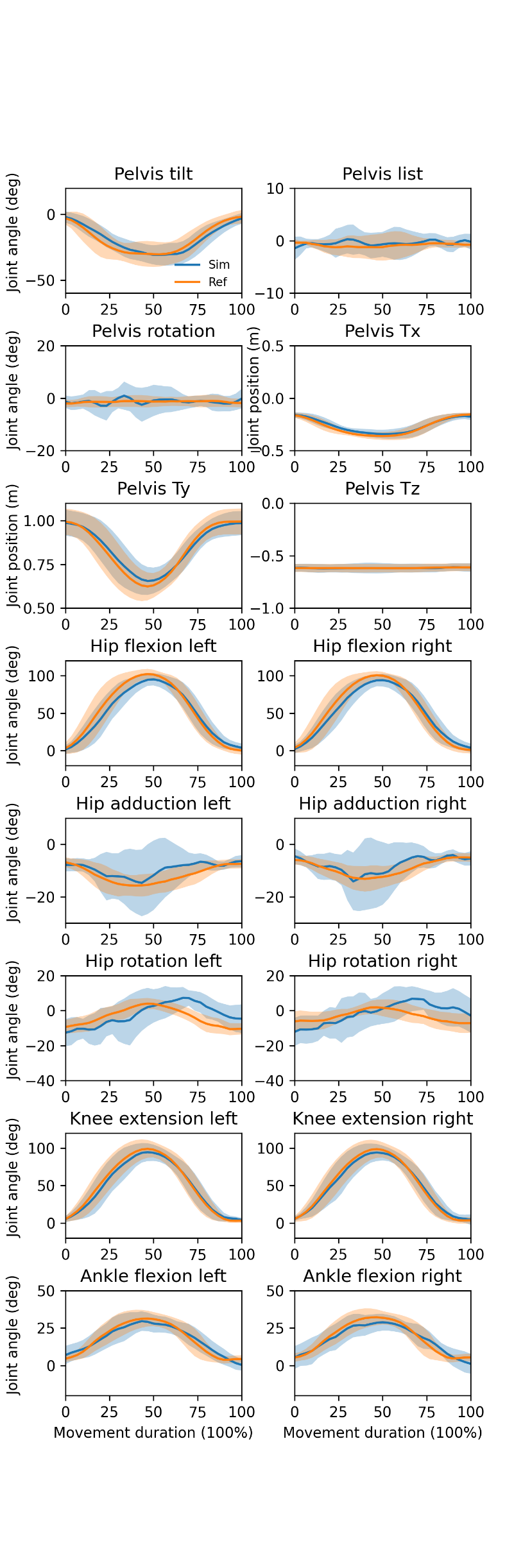


Fig S2-3. Reference kinematics in the squatting task and kinematics tracked using direct collocation method (Gaussian noise with default setting)


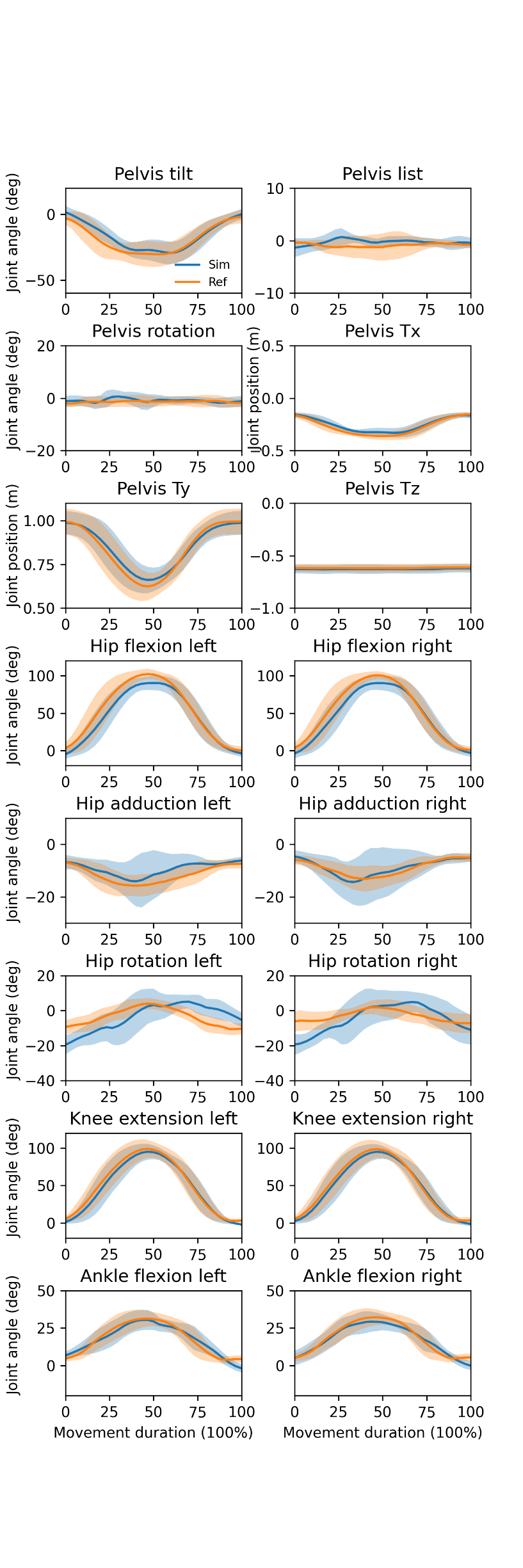


Fig S2-4. Reference kinematics in the squatting task and kinematics tracked using direct collocation method (Noisy group1 level with default setting)


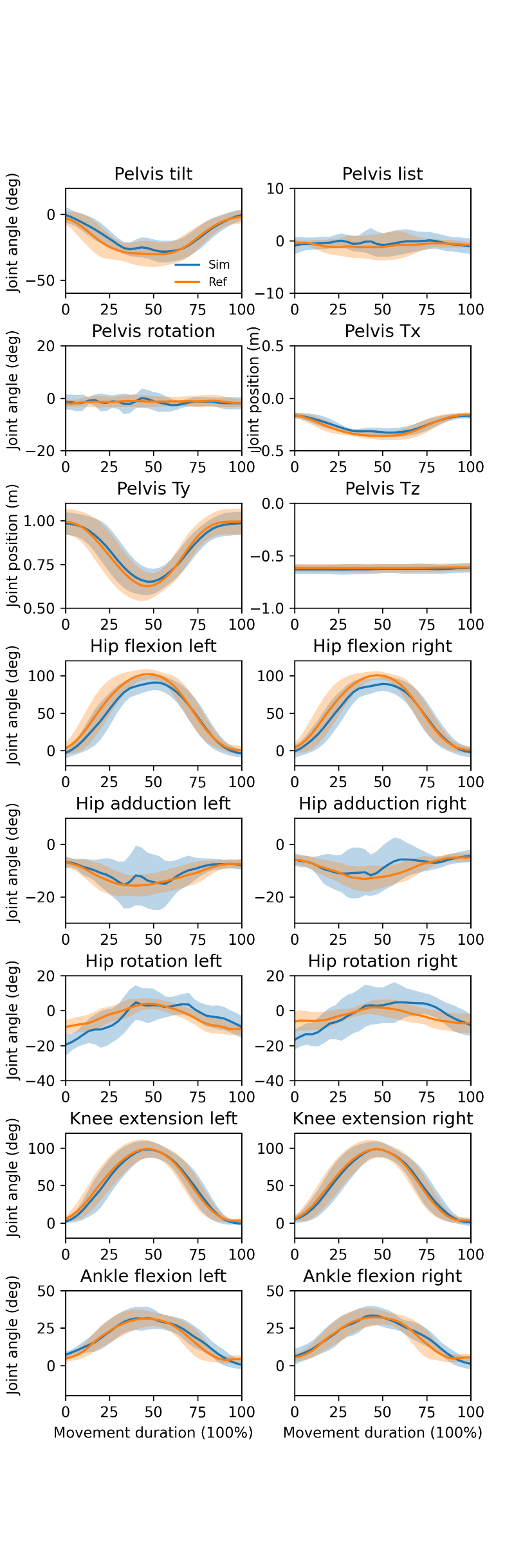


Fig S2-5. Reference kinematics in the squatting task and kinematics tracked using direct collocation method (Noisy group2 level with default setting)


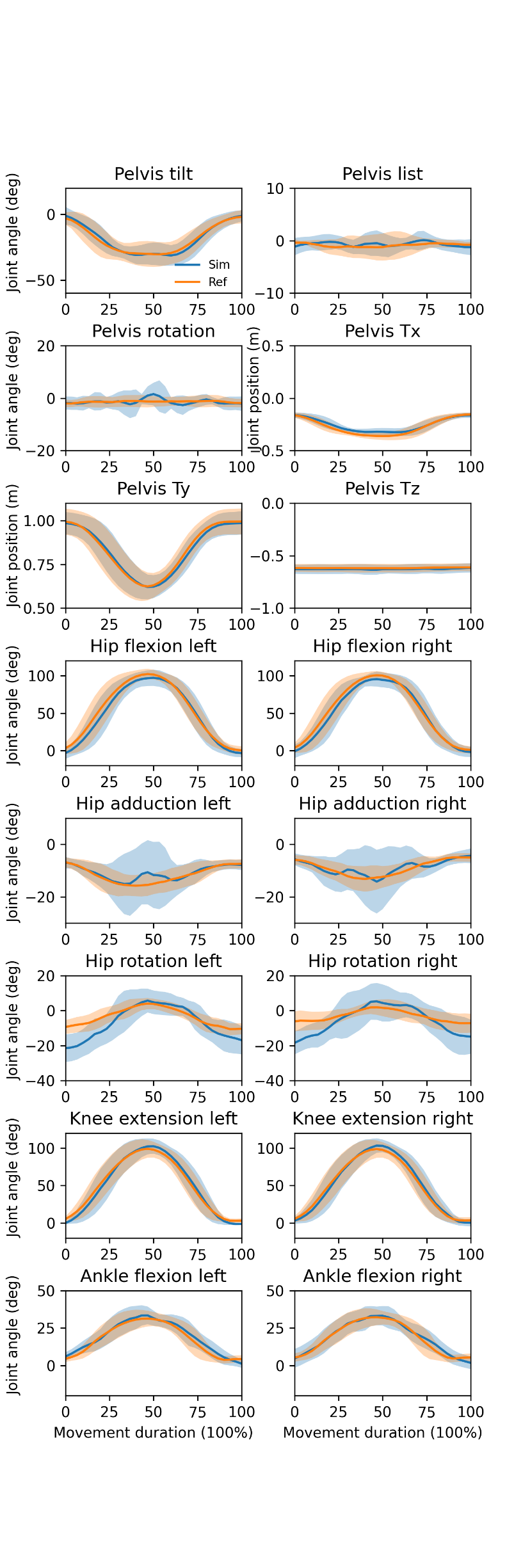


Fig S2-6. Reference kinematics in the squatting task and kinematics tracked using direct collocation method (Noisy group2 level with zero metabolic weighting (M0))


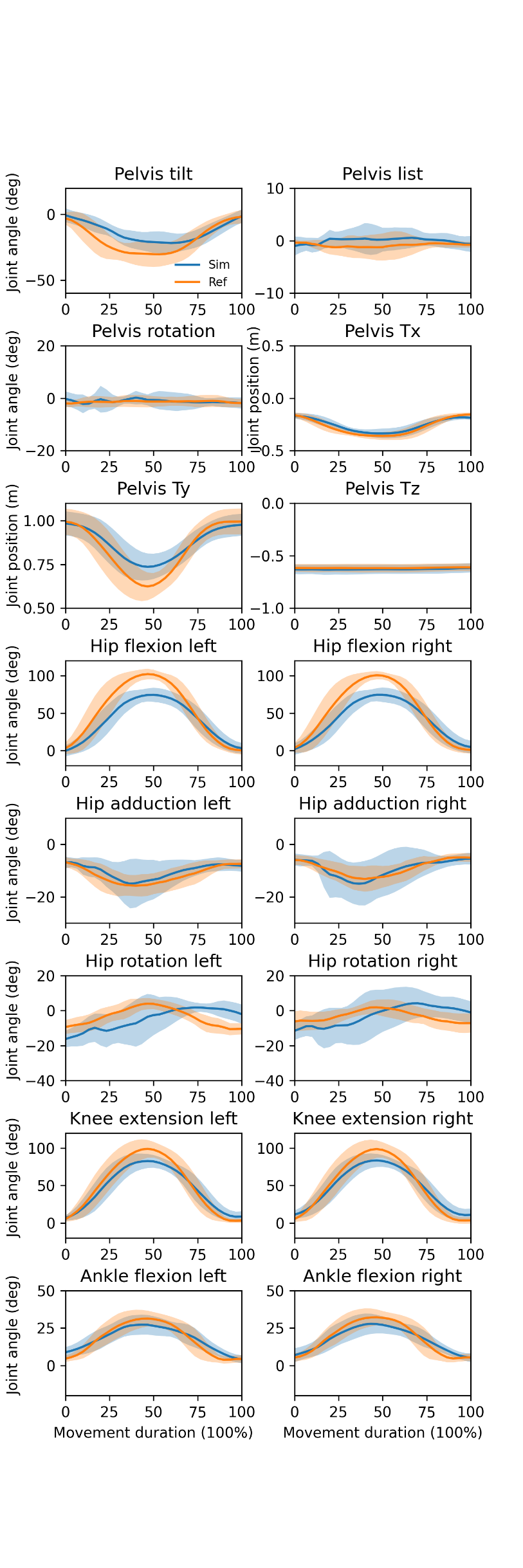


Fig S2-7. Reference kinematics in the squatting task and kinematics tracked using direct collocation method (Noisy group2 level with ten times of metabolic weighting (M10))


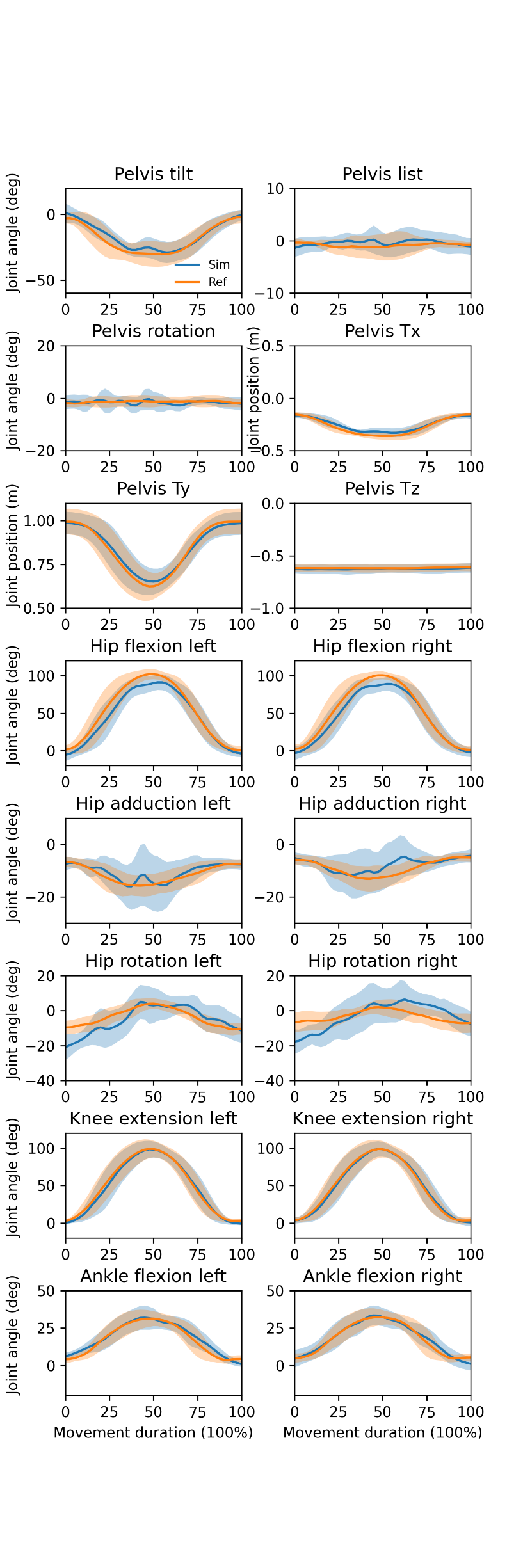


Fig S2-8. Reference kinematics in the squatting task and kinematics tracked using direct collocation method (Noisy group2 level with 40 mesh intervals (N40))


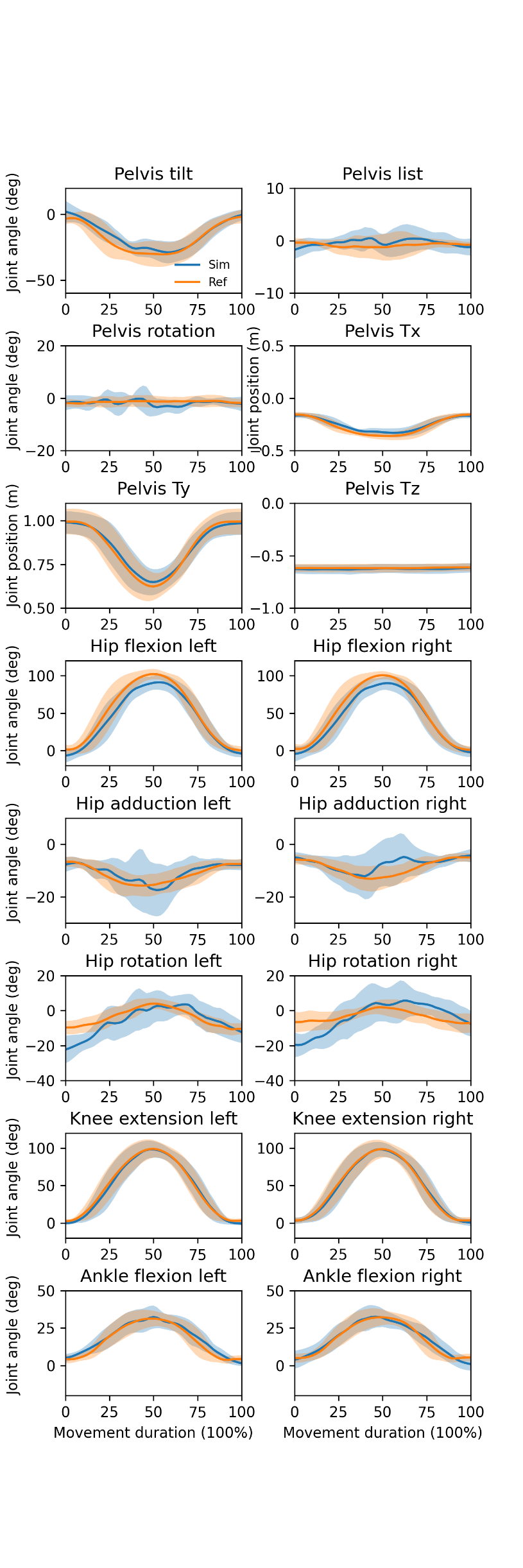


Fig S2-9. Reference kinematics in the squatting task and kinematics tracked using direct collocation method (Noisy group2 level with 50 mesh intervals (N50))
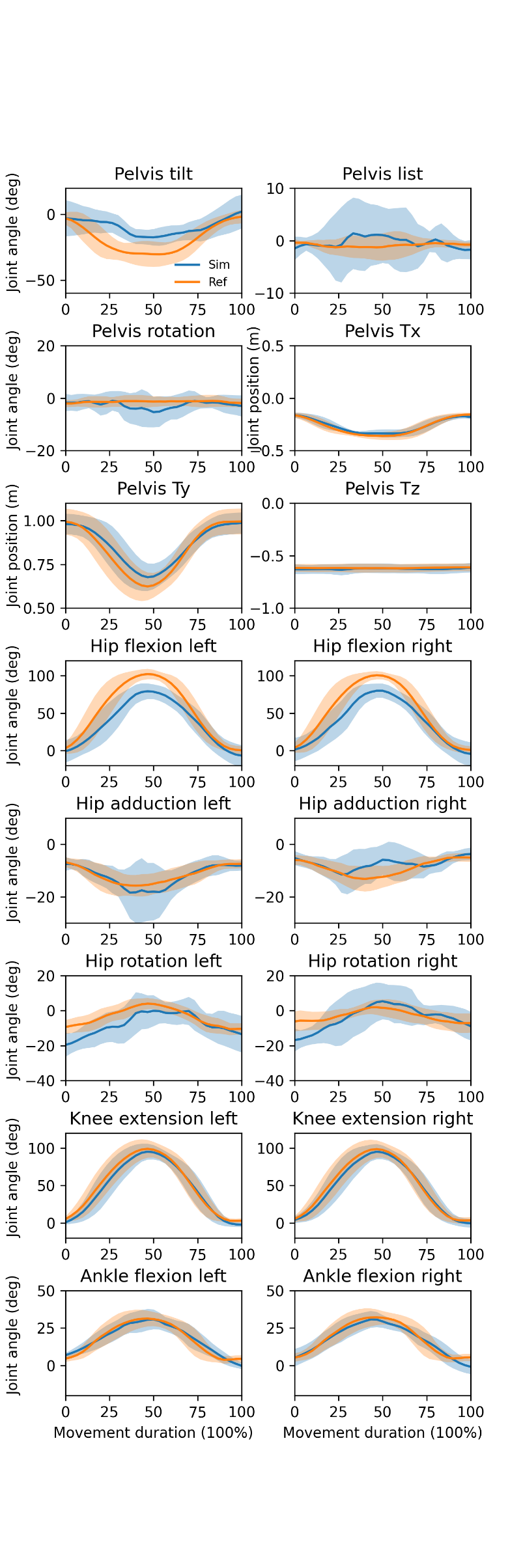


Fig S2-10. Reference kinematics in the squatting task and kinematics tracked using direct collocation method (Noisy group2 level with zero passive torque weighting (P0))
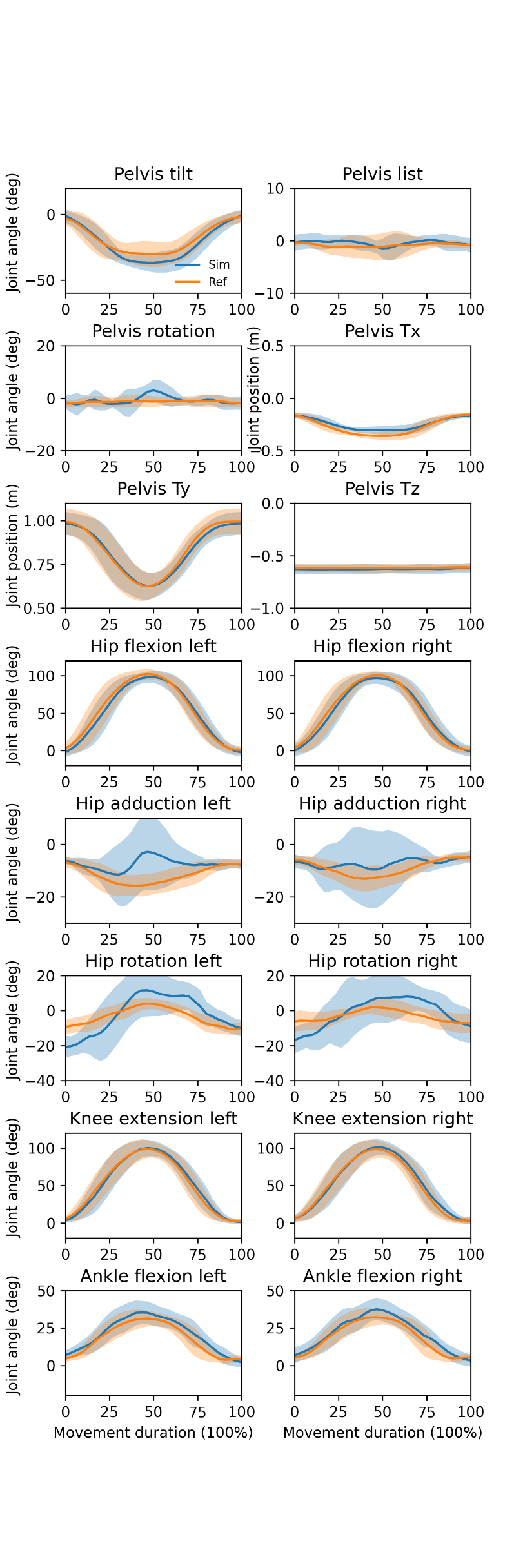


Fig S2-11. Reference kinematics in the squatting task and kinematics tracked using direct collocation method (Noisy group2 level with ten times of passive torque weighting (P10))


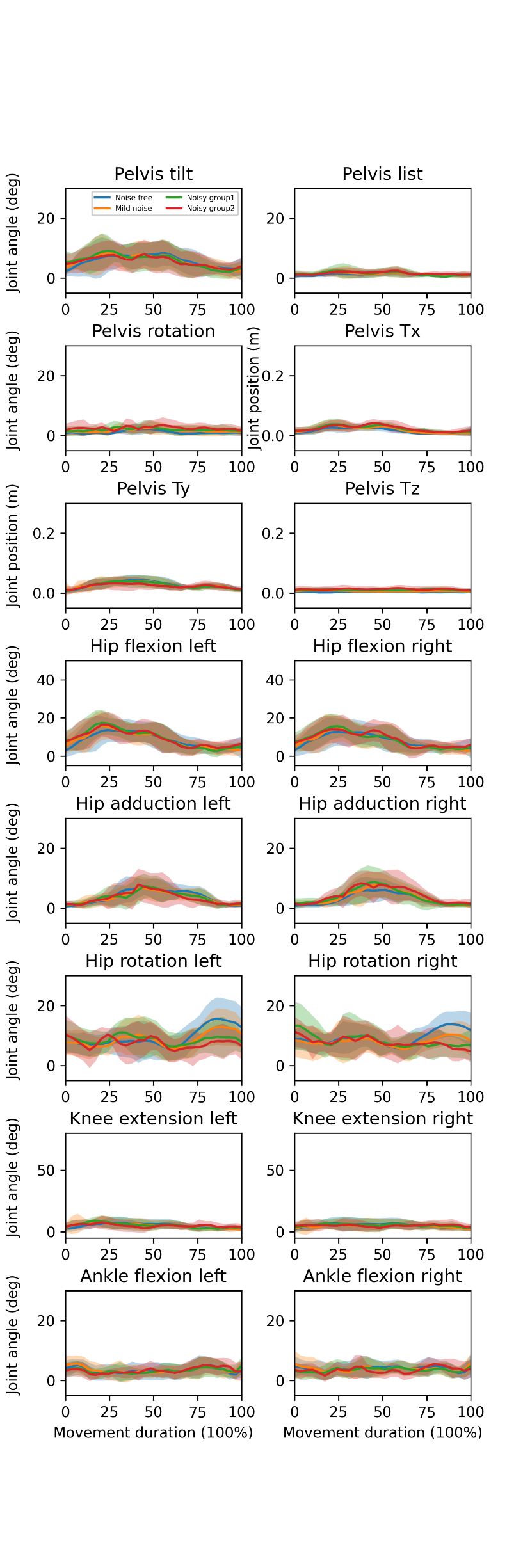


Fig S2-12. Mean absolute errors and standard deviations in the squatting task between reference kinematics and kinematics tracked using direct collocation method
