# Supplementary material for: Robust kinetics estimation from kinematics via direct collocation
Source: Front Bioeng Biotechnol. 2024 Dec 18;12:1483225. doi: 10.3389/fbioe.2024.1483225 (PMC11688375; doi:10.3389/fbioe.2024.1483225)
Supplement: Supplementary file 6 [file DataSheet1.docx]

Supplemental material 1


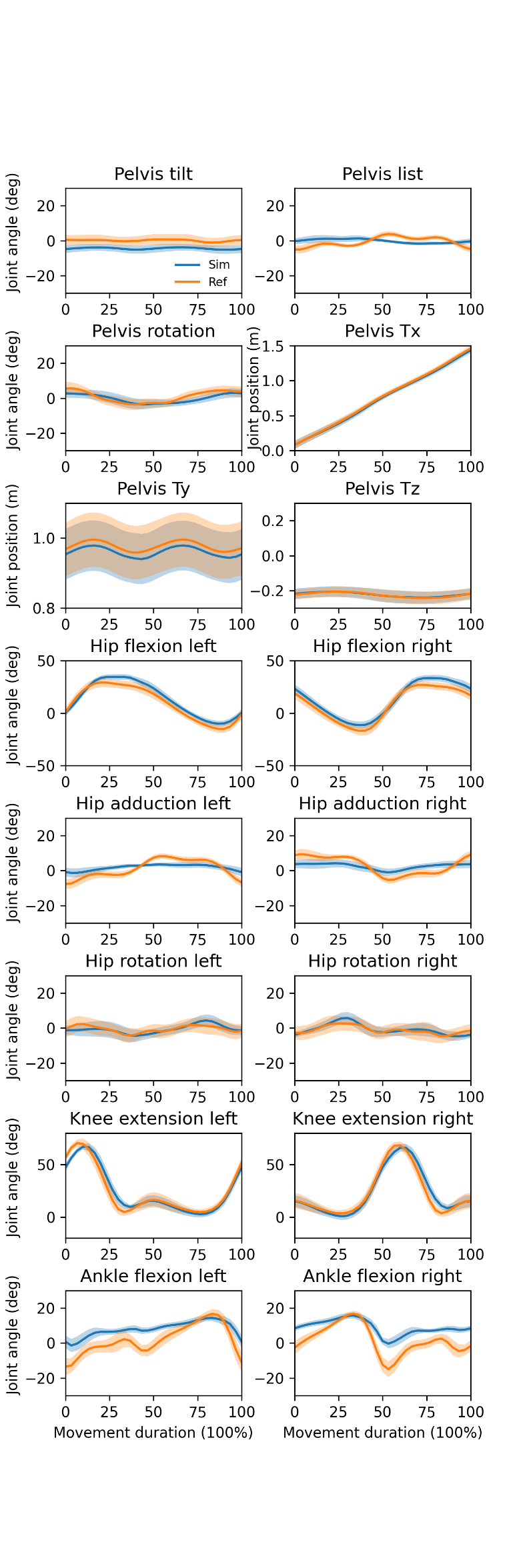


Fig S1-1. Reference kinematics in the walking task and kinematics tracked using direct collocation method (Noise free level with default setting)


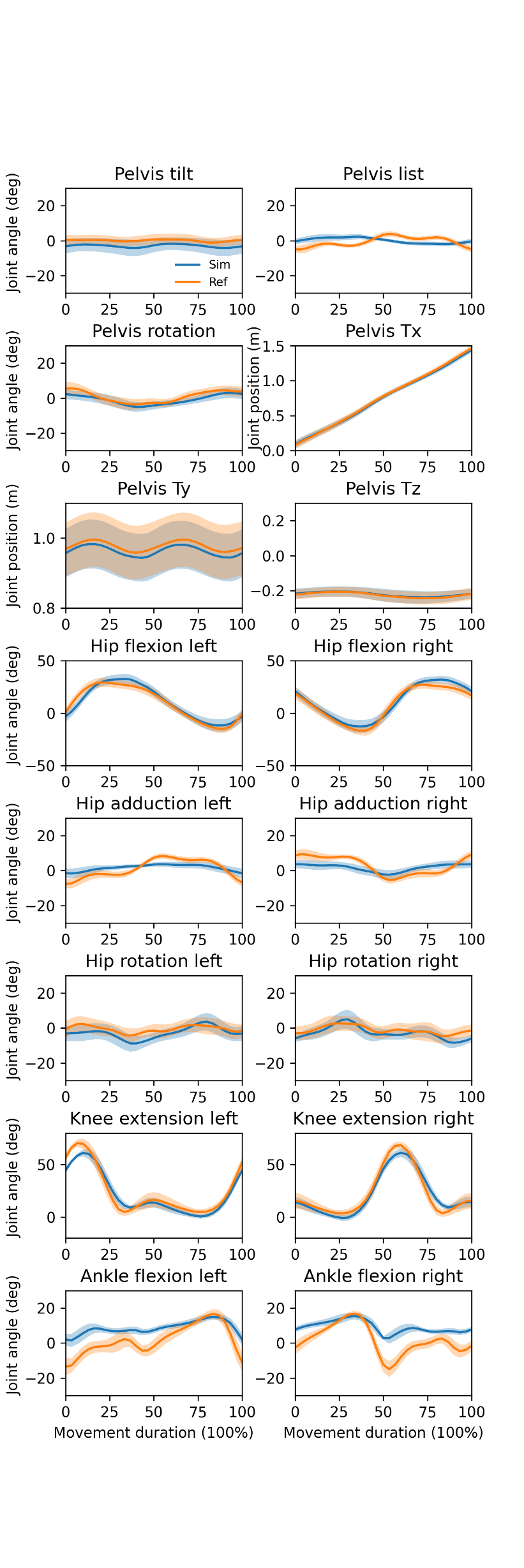


Fig S1-2. Reference kinematics in the walking task and kinematics tracked using direct collocation method (Mild noise level with default setting)


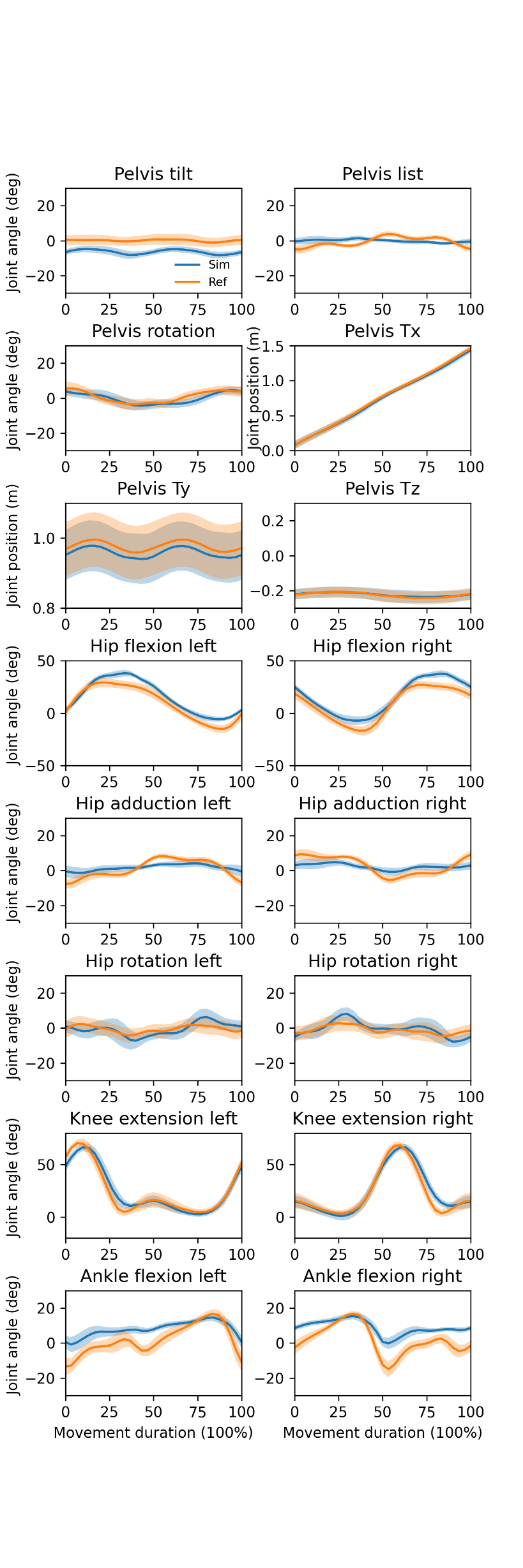


Fig S1-3. Reference kinematics in the walking task and kinematics tracked using direct collocation method (Gaussian noise with default setting)


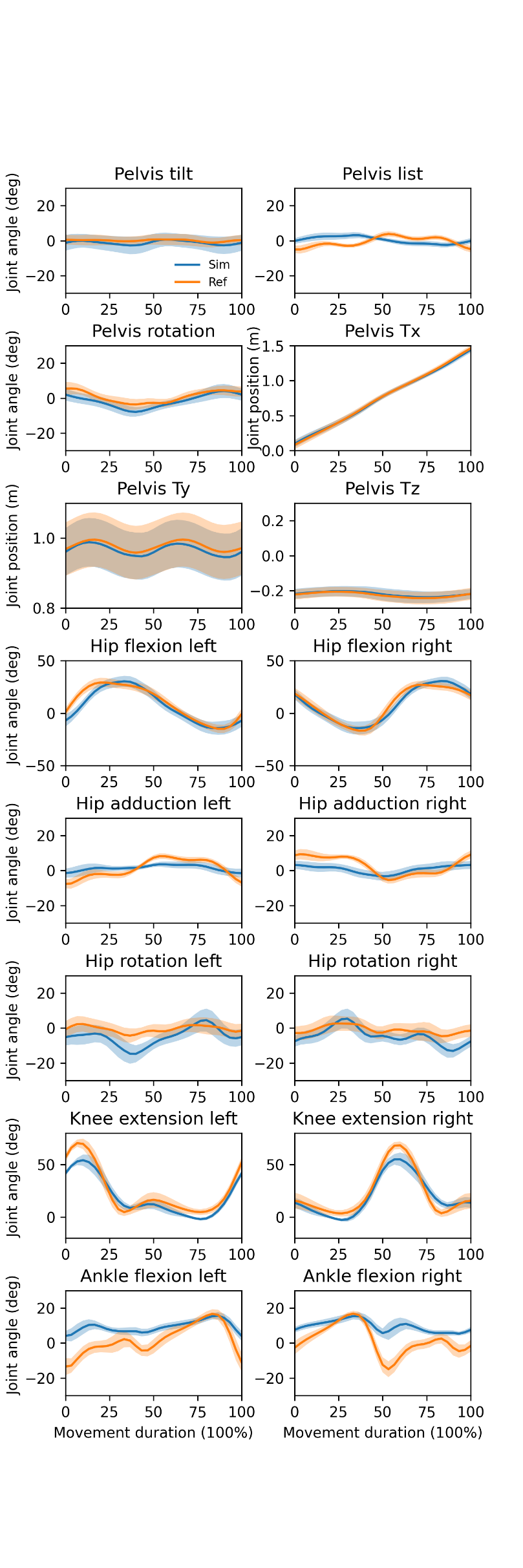


Fig S1-4. Reference kinematics in the walking task and kinematics tracked using direct collocation method (Noisy group1 level with default setting)


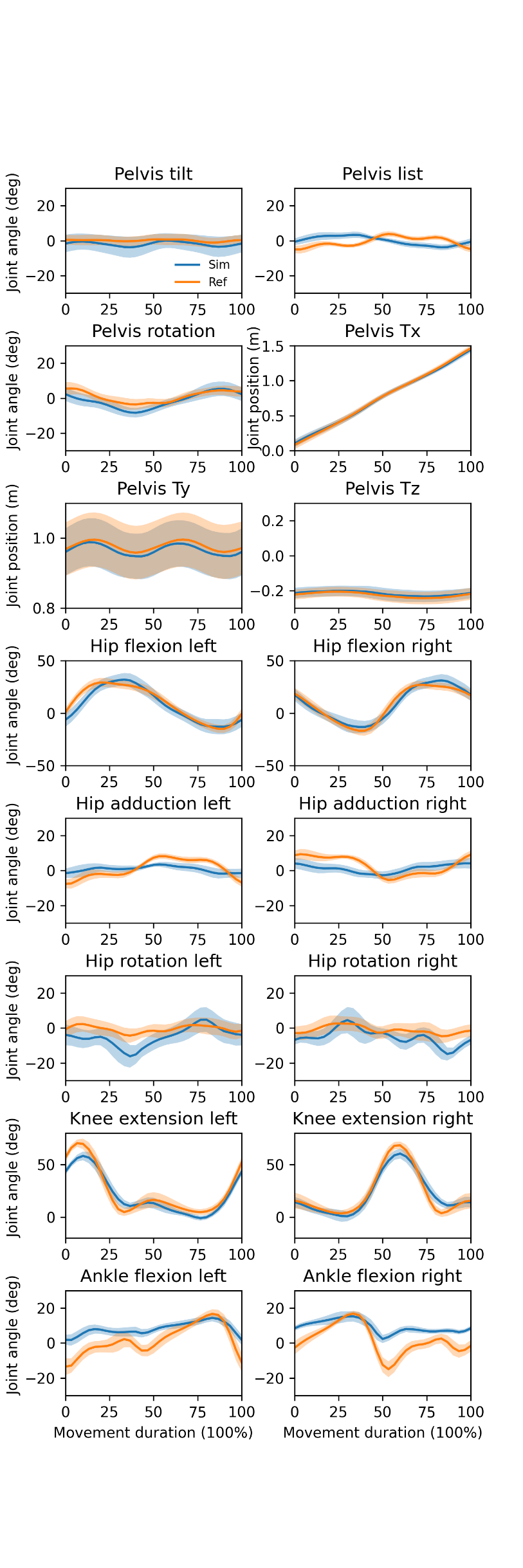


Fig S1-5. Reference kinematics in the walking task and kinematics tracked using direct collocation method (Noisy group2 level with default setting)


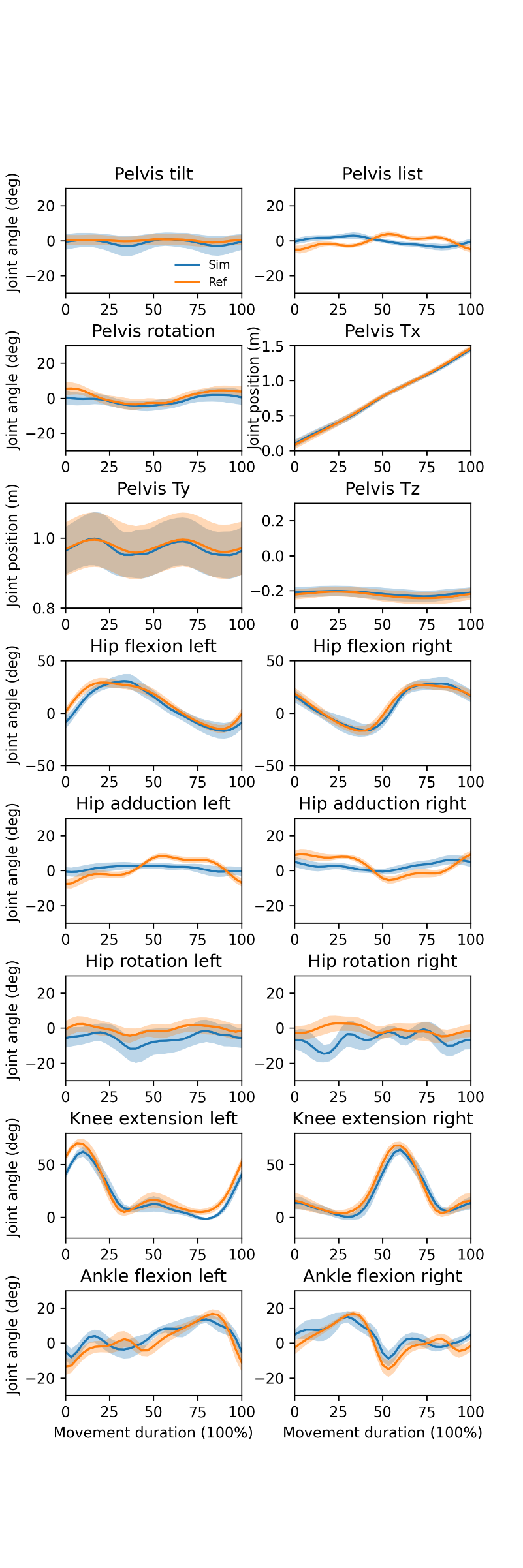


Fig S1-6. Reference kinematics in the walking task and kinematics tracked using direct collocation method (Noisy group2 level with zero metabolic weighting (M0))


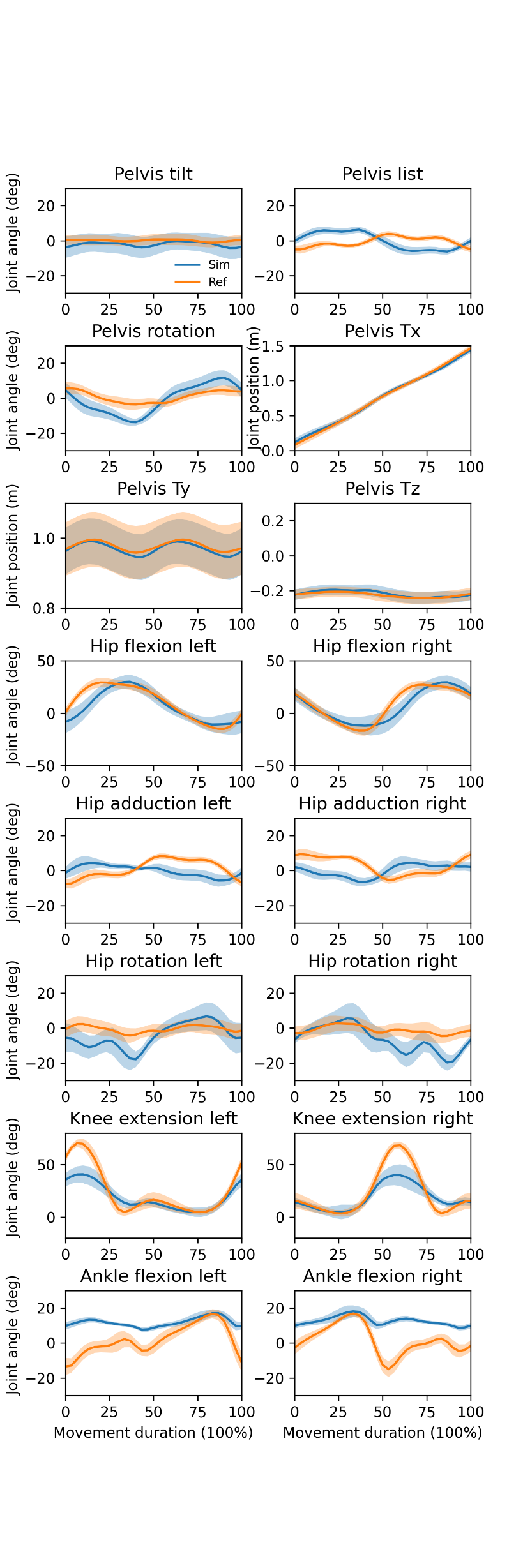


Fig S1-7. Reference kinematics in the walking task and kinematics tracked using direct collocation method (Noisy group2 level with ten times of metabolic weighting (M10))


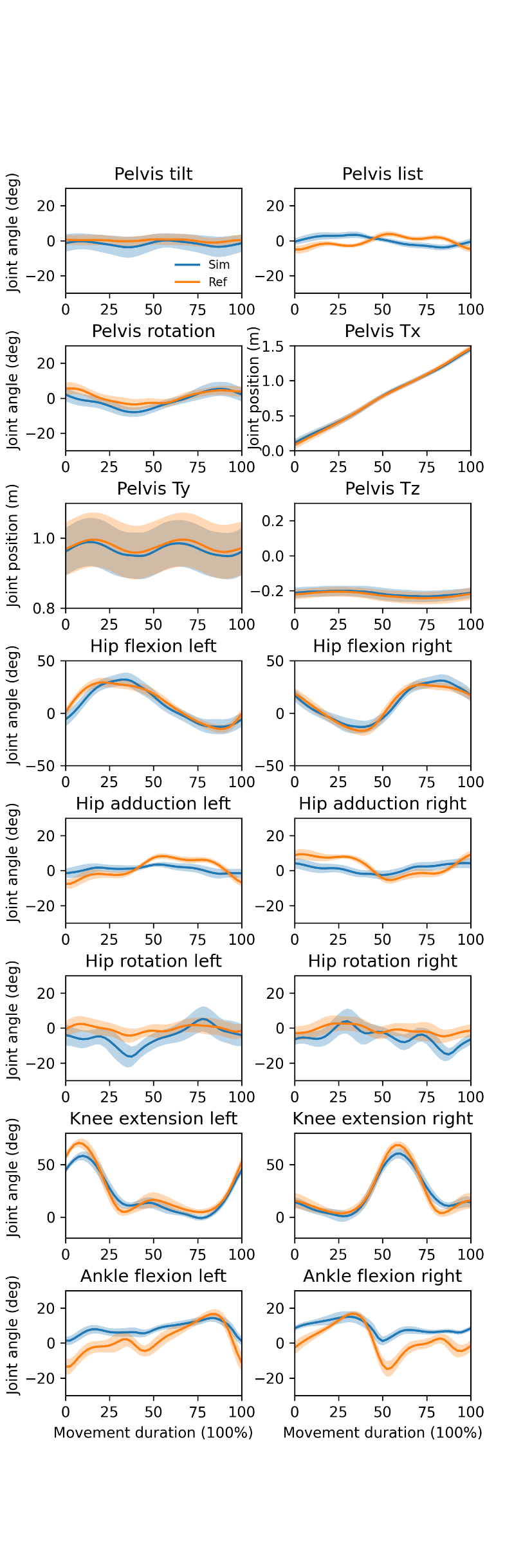


Fig S1-8. Reference kinematics in the walking task and kinematics tracked using direct collocation method (Noisy group2 level with 40 mesh intervals (N40))


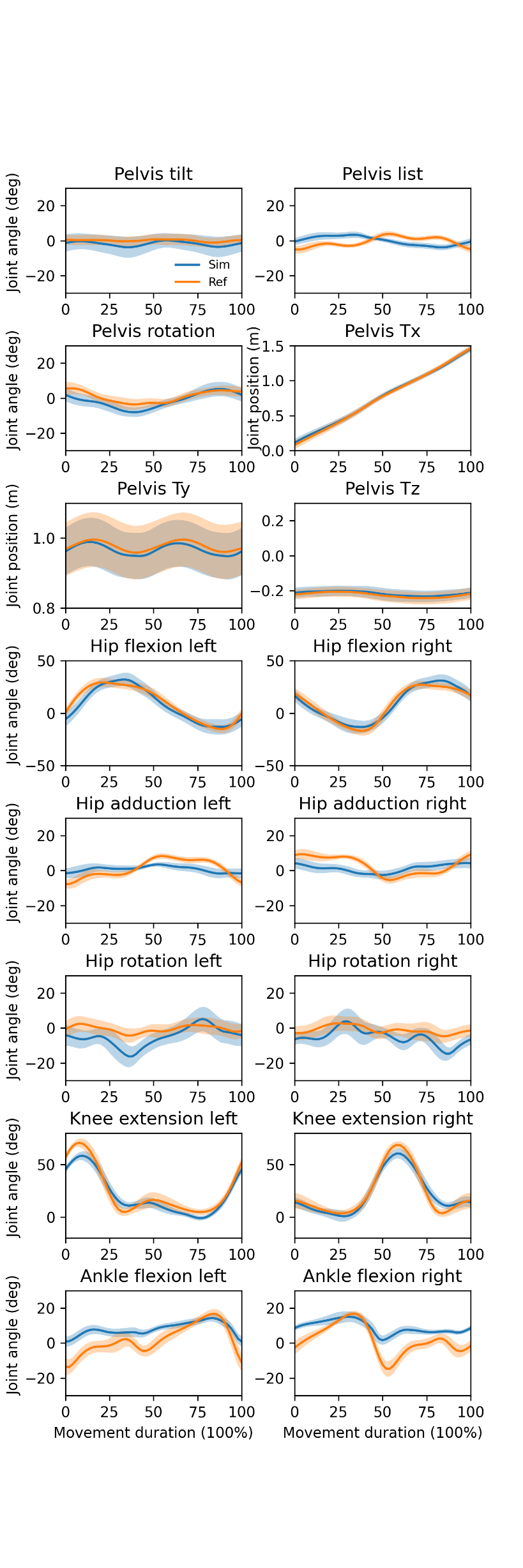


Fig S1-9. Reference kinematics in the walking task and kinematics tracked using direct collocation method (Noisy group2 level with 50 mesh intervals (N50))


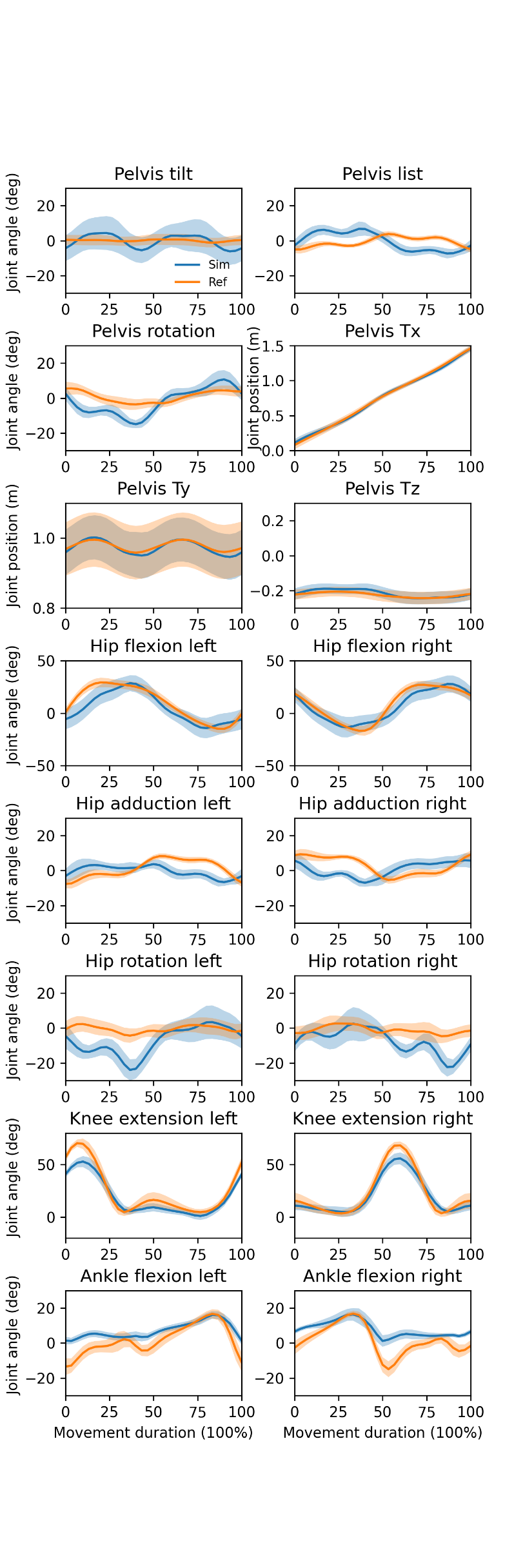


Fig S1-10. Reference kinematics in the walking task and kinematics tracked using direct collocation method (Noisy group2 level with zero passive torque weighting (P0))


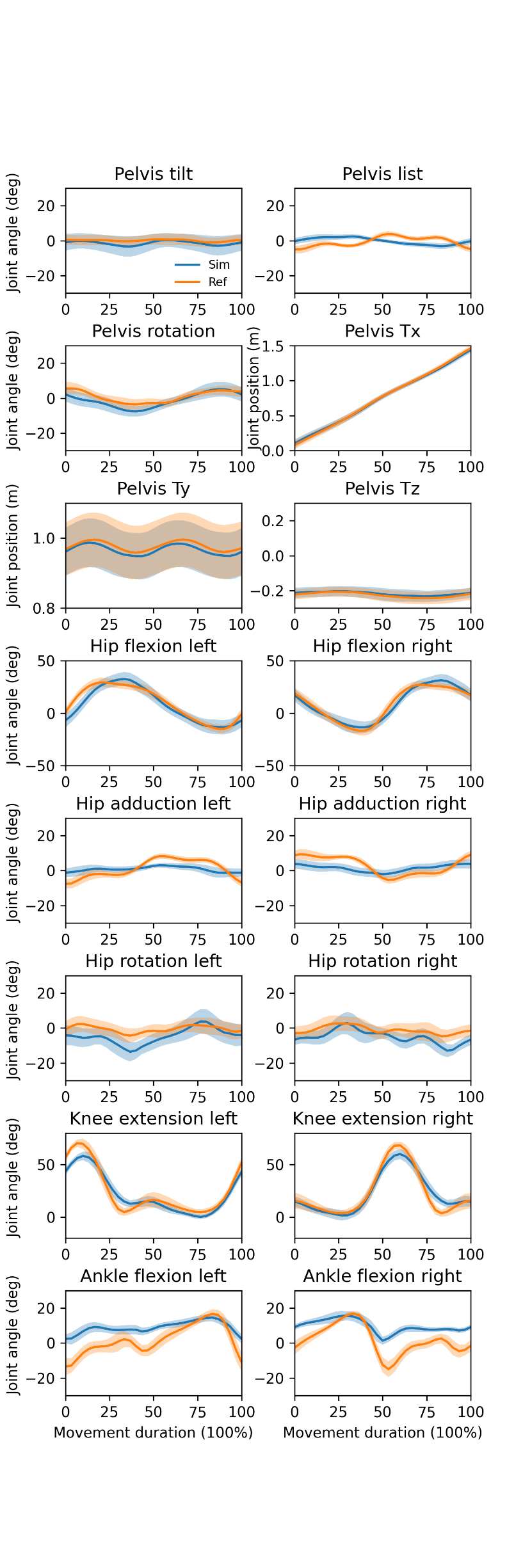


Fig S1-11. Reference kinematics in the walking task and kinematics tracked using direct collocation method (Noisy group2 level with ten times of passive torque weighting (P10))


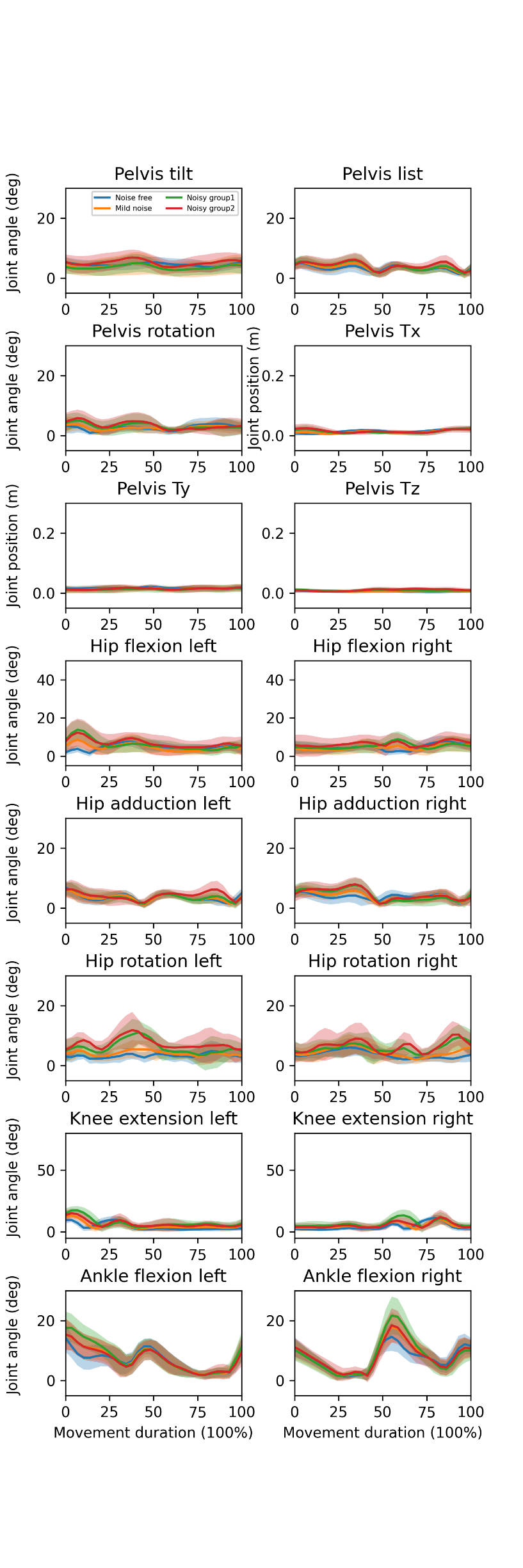


Fig S1-12. Mean absolute errors and standard deviations in the walking task between reference kinematics and kinematics tracked using direct collocation method
